# A visualized machine learning model using noninvasive parameters to differentiate men with and without prostatic carcinoma before biopsy

**DOI:** 10.1038/s41598-025-12765-2

**Published:** 2025-07-27

**Authors:** Wenting Zhou, Linhui Wang, Xue Zhang, Xiaohong Zou, Xuemei Du, Liru Luo, Xiaolan Ye, Shujing Li, Hong Lv, Yuanfu Liu, Xiaoyang Huang

**Affiliations:** 1 Department of Pathology, The First People’s Hospital of Longquanyi District, Chengdu, 610100 China; 2 Department of General Practice, Tong’an Community Health Service Center in Longquanyi District, Chengdu, China; 3 Department of Urinary and Neohropathy Center, Second Affiliated Hospital of Chongqing Medical University, Chongqing, China; 4 Shenzhen, 518110 China

**Keywords:** Prostatic neoplasms, Prostatic hyperplasia, Thymidine kinase 1, Risk assessment, Machine learning, Cancer, Computational biology and bioinformatics, Medical research, Oncology, Prostate

## Abstract

This study aimed to create a visualized extreme gradient boosting (XGBOOST) model to distinguish prostatic carcinoma (PCA) from non-PCA using noninvasive prebiopsy parameters before biopsy. This was a cross-sectional study of 310 Chinese men who underwent prostate biopsy and were divided into PCA (*n* = 126) and non-PCA (*n* = 184) groups. The non-PCA patients were diagnosed with benign prostatic hyperplasia (BPH) based on biopsy results. The XGBOOST model was used to analyze 15 noninvasive prebiopsy parameters. The model performance was assessed by the area under the receiver operating characteristic curve (AUC) and compared with four other machine learning models (decision tree learning, lasso, neural network (NNET), and support vector machine (SVM)) and a logistic model. The logistic model identified serum thymidine kinase 1 (STK1p), total prostate-specific antigen (TPSA), and age as key prognostic factors. In the Lasso procedure, free prostate-specific antigen (FPSA) and free-to-total prostate-specific antigen (FTPSA) were also added to machine learning models. The XGBOOST model achieved an AUC of 0.965, which was significantly greater than those of other models (AUC = 0.708–0.817) and the logistic model (AUC = 0.813) (*P* < 0.001). The 49 decision trees generated by the XGBOOST model were visualized to aid in decision making. This study successfully developed a visualized XGBOOST model with high accuracy in differentiating PCA from non-PCA using eight noninvasive predictors. This model could aid in the precise selection of high-risk PCA patients for biopsy, potentially minimizing unnecessary procedures and their associated costs.

**Supplementary Information:**

The online version contains supplementary material available at 10.1038/s41598-025-12765-2.

## Introduction

Prostatic carcinoma (PCA) is one of the most common cancers and is a major burden for men, with an estimated 0.4 million deaths worldwide in 2020^1^. In China, PCA had the 6th highest incidence and 7th highest mortality among male cancers in 2020. PCA is characterized by an asymptomatic onset and slow progression, so most patients have late diagnoses and poor prognoses^2^. Screening is an important way to identify early cases of PCA.

Currently, China’s PCA screening guidelines recommend the use of total prostate-specific antigen (TPSA) for primary screening^3^. However, a recent meta-analysis based on a Chinese population reported that benign diseases such as benign prostatic hyperplasia (BPH) were also related to elevated levels of TPSA. Therefore, this indicator has high sensitivity (pooled estimate: 91%) but low specificity (41%)^4^. A meta-analysis including 19 studies from different countries also reported that the TPSA had a high sensitivity (pooled estimate: 93%) but a low specificity (20%)^5^. This may lead to patient anxiety, increased costs, and potential harm related to unnecessary biopsies. A recent systematic review reported that most PCA models had good performance, with a pooled area under the receiver operating characteristic curve (AUC) of 0.78 (95% CI: 0.73, 0.82) (I^2^ = 96%)^6^. In routine care in China, patients who have enlarged prostate glands identified through digital rectal examination or ultrasound images with moderate or severe lower urinary tract symptoms are usually referred for prostate biopsy. However, few current studies have reported models specific for patients who often experience a more significant impact on their quality of life than for those with mild symptoms^7^. Precisely assessing PCA risk in this group is important for addressing immediate concerns and reducing unnecessary biopsies.

Thymidine kinase 1 (TK1) is recognized as a cell proliferation biomarker that has potential value in cancer risk assessment^8–10^. A recent study showed that the serum TK1 protein (STK1p) concentration is associated with PCA^11^. A model involving STK1p may help to improve the accuracy of PCA risk assessment. Furthermore, the use of machine learning for the classification and prediction of disease outcomes has been shown to be highly efficient in the field of clinical research^12^. Therefore, this study aimed to develop a machine learning model using STK1p and other noninvasive predictors for classifying PCA and BPH to help precisely select high-risk patients for biopsy.

## Methods

### Dataset preparation

**Fig. 1:**
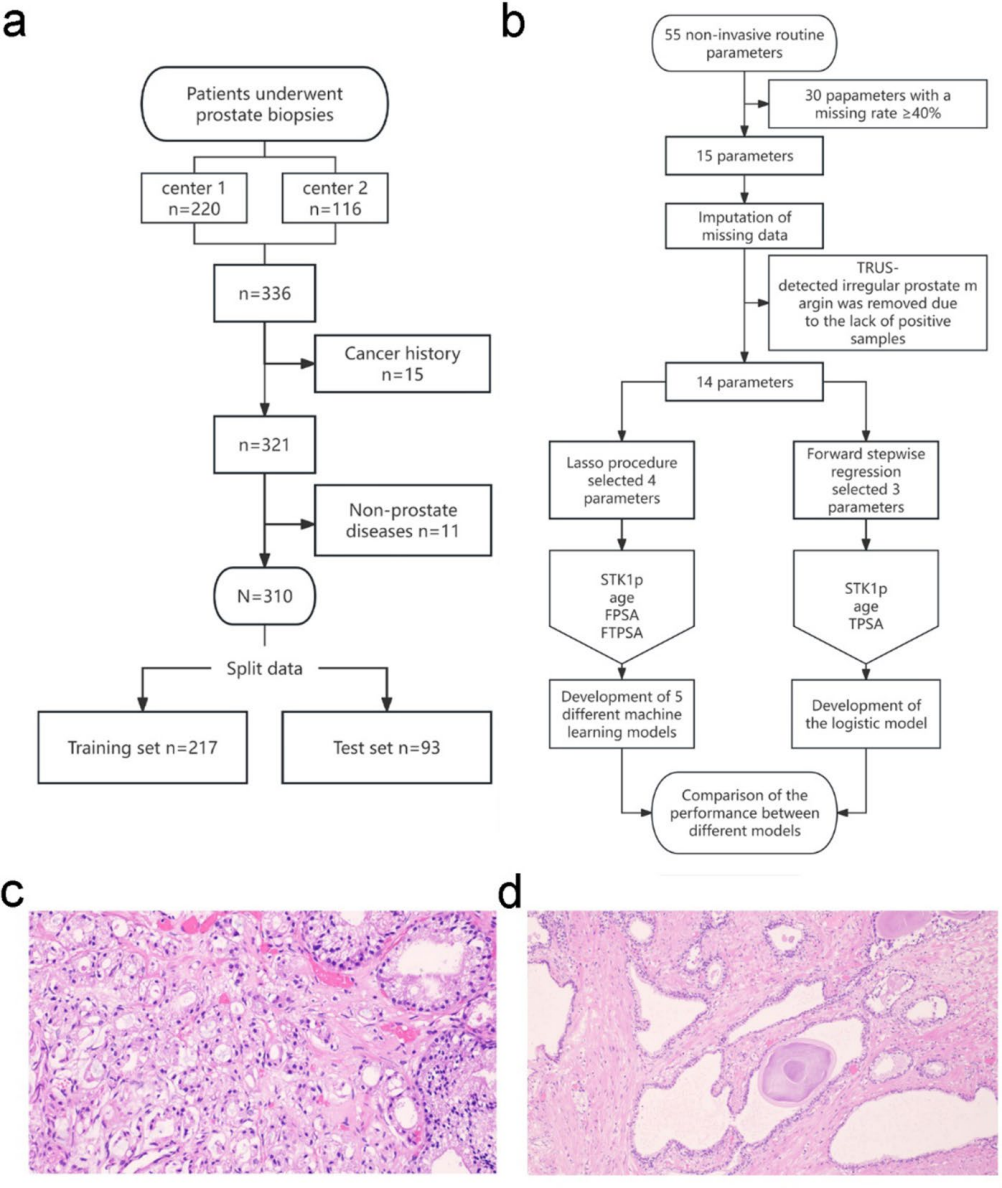
(**a**) Flowchart of the study participants. Center 1 refers to the First People’s Hospital of Longquanyi District; Center 2 refers to the Daping Hospital of the Third Military Medical University. The Gleason scores of the prostatic carcinoma patients were between 6 and 9. (**b**) The procedure for developing machine learning models and a logistic model. Histology images of prostatic carcinoma (**c**) and benign prostatic hyperplasia (d) from the study participants.

This study used a cross-sectional design. Male patients with suspected PCA who underwent prostate biopsies (Fig. 1a) at the First People’s Hospital of Longquanyi District, China, from June 1, 2022, to April 1, 2024, and at the Daping Hospital of the Third Military Medical University, China, from May 1, 2016, to September 1, 2017, were considered eligible. Patients with a history of cancer diagnosis prior to biopsy were excluded. Clinical methods were carried out in accordance with the Guidelines for the Diagnosis and Treatment of Prostate Cancer by the Chinese Society of Clinical Oncology. The requirement to obtain informed consent for inclusion in this analysis was waived by the Medical Ethics Committee of First People’s Hospital of Longquanyi District, Chengdu (No. AF-KY-2021002).

Noninvasive parameters measured at inpatient departments within one day before biopsy were included as candidate predictors in this study. These parameters were chosen based on a thorough review of the existing literature, which suggested their potential relevance to the pathophysiology of PCA and could be measured using non-invasive, well-established, and validated methods. Parameters with a missing data rate of less than 40% were included. These included age, irregular margin (TRUS) parameters, and 13 serum biomarkers.

The 13 serum biomarkers included STK1p, TPSA, free prostate-specific antigen (FPSA), free/total prostate-specific antigen ratio (FTPSA), alpha-fetoprotein (AFP), carcinoembryonic antigen (CEA), serum ferritin (SF), neuron-specific enolase (NSE), β-human chorionic gonadotropin (β-HCG), cancer antigen 125 (CA125), cancer antigen 15 − 3 (CA15-3), cancer antigen 72 − 4 (CA72-4), and cancer antigen 19 − 9 (CA19-9). STK1p was detected by a TK1 antibody-based enhanced chemiluminescence dot blot and analyzer, which was validated in previous research^8^. Other serum biomarkers were detected by a chemiluminescence dot blot and analyzer (Cobas 1601 auto analyzer of Roche, Switzerland).

Two senior pathologists confirmed the biopsy results according to the Guidelines for the Diagnosis and Treatment of Prostate Cancer by the Chinese Society of Clinical Oncology. Patients with a positive biopsy result (PCA) were referred for prostate surgery and further pathological tests. Examples of histology images of PCA and BPH from the study participants are presented in Fig. 1c and d, respectively.

### Model development and validation

The Lasso logistic algorithm was also used to select significant predictors with 10-fold cross-validation for overfitting reduction (Fig. 1b). The predictors with the minimum cross-validation mean variance selected by the Lasso procedure were used to develop machine learning models. Five machine learning methods, namely, extreme gradient boosting (XGBOOST), decision tree learning, lasso, neural network (NNET), and support vector machine (SVM), were used to model PCA. The XGBoost model was optimized using a grid search approach with the following hyperparameters: learning rate (0.1), maximum depth of trees (5), and subsample ratio (0.8). The mean Shapley additive explanations (SHAP) approach was used to measure the importance of each candidate predictor.

Forward stepwise selection was used to screen candidate predictors and fit the logistic model. The odds ratio (OR), 95% confidence interval (CI), and logistic regression coefficients were calculated for each selected predictor.

The dataset of 310 patients was randomly split into training (70%) and test (30%) sets to evaluate the model performance (Table 1). To ensure the models’ stability and generalizability, 10-fold cross-validation was employed during the training phase. This process involved partitioning the training data into 10 equal parts, with each part serving as the validation set once while the remaining nine parts were used for training. The AUC of each model was calculated to assess its performance. The cutoff point for the logistic and lasso models was identified based on the highest specificity when the sensitivity was ≥ 91% for comparison with the TPSA^4^. The net benefits of machine learning models were compared with those of logistic models. The net benefit was analyzed using a decision curve to assist in the clinical trade-off between the potential benefits (e.g., PCA diagnosis) and harms (e.g., unnecessary biopsies) of a method.

**Table 1:** Characteristics of the study participants.

Characteristics		Non-prostatic carcinoma	Prostatic carcinoma	*P* (Wilcoxon Rank Sum Test)	Overall	
**Overall set**						
n		184	126		310	
Age (year)		70 [66, 75]	73 [68, 79]	0.001	72 [67, 77]	
Irregular prostate margin		3 (1.63)	18 (14.29)	< 0.001	21 (6.77)	
STK1p (pmol/L)		0.84 [0.30, 1.53]	2.04 [1.05, 3.03]	< 0.001	1.09 [0.56, 2.20]	
TPSA (µg/L)		5.70 [2.16, 13.49]	11.38 [1.55, 78.93]	0.020	6.77 [1.82, 18.70]	
FPSA (µg/L)		0.99 [0.44, 1.91]	1.69 [0.54, 5.00]	0.001	1.16 [0.46, 2.59]	
FTPSA		0.19 [0.11, 0.26]	0.11 [0.06, 0.35]	0.013	0.16 [0.09, 0.27]	
NSE (µg/L)		10.70 [8.78, 13.60]	11.00 [8.80, 14.20]	0.657	10.90 [8.80, 13.70]	
AFP (µg/L)		3.05 [2.20, 4.20]	2.75 [2.05, 3.70]	0.065	3.00 [2.20, 3.80]	
CEA (µg/L)		2.37 [1.88, 3.49]	2.49 [1.98, 3.17]	0.631	2.47 [1.93, 3.39]	
SF (µg/L)		350.00 [235.50, 528.00]	331.00 [193.50, 528.00]	0.472	344.00 [216.00, 528.00]	
β-HCG (U/L)		0.10 [0.10, 0.21]	0.10 [0.10, 0.24]	0.570	0.10 [0.10, 0.21]	
CA125 (kU/L)		13.41 [9.86, 18.10]	11.82 [8.72, 17.10]	0.078	12.19 [9.67, 17.47]	
CA15-3 (kU/L)		11.73 [8.88, 15.91]	12.30 [8.64, 15.65]	0.579	12.06 [8.65, 15.91]	
CA72-4 (kU/L)		3.05 [1.45, 8.12]	2.62 [1.55, 6.10]	0.625	2.94 [1.48, 6.98]	
CA19-9 (kU/L)		10.70 [5.78, 20.49]	11.12 [6.65, 20.60]	0.245	11.02 [5.78, 20.60]	
**Training set**						
n		129	88		217	
Age (year)		70 [66, 75]	73 [68, 79]	0.002	72 [67, 77]	
STK1p (pmol/L)		0.81 [0.29, 1.49]	2.10 [1.05, 3.21]	< 0.001	1.10 [0.53, 2.20]	
TPSA (µg/L)		6.24 [2.38, 13.90]	13.10 [1.59, 68.99]	0.059	7.23 [2.07, 20.91]	
FPSA (µg/L)		1.09 [0.44, 1.94]	1.72 [0.56, 5.00]	0.002	1.22 [0.47, 2.59]	
FTPSA		0.18 [0.11, 0.25]	0.12 [0.06, 0.36]	0.112	0.16 [0.09, 0.27]	
NSE (µg/L)		10.70 [8.80, 13.70]	11.90 [8.80, 14.22]	0.463	11.00 [8.80, 14.00]	
AFP (µg/L)		3.10 [2.20, 3.80]	2.65 [2.20, 3.40]	0.075	2.90 [2.20, 3.70]	
CEA (µg/L)		2.51 [1.93, 3.76]	2.53 [2.06, 3.22]	0.982	2.51 [1.94, 3.66]	
SF (µg/L)		341.00 [229.00, 512.00]	340.50 [200.00, 525.75]	0.962	341.00 [216.00, 523.00]	
β-HCG (U/L)		0.10 [0.10, 0.21]	0.10 [0.10, 0.22]	0.670	0.10 [0.10, 0.21]	
CA125 (kU/L)		13.47 [9.86, 18.10]	11.98 [9.50, 17.25]	0.362	12.72 [9.67, 17.49]	
CA15-3 (kU/L)		12.32 [9.42, 16.42]	11.91 [8.63, 15.03]	0.102	12.06 [8.65, 15.91]	
CA72-4 (kU/L)		2.86 [1.37, 6.98]	2.57 [1.37, 5.35]	0.810	2.63 [1.37, 6.10]	
CA19-9 (kU/L)		10.70 [5.78, 20.44]	12.63 [7.75, 20.49]	0.100	11.07 [6.46, 20.44]	
**Test set**						
n		55	38		93	
Age (year)		70 [67, 75]	73 [68, 78]	0.258	72 [67, 76]	
STK1p (pmol/L)		0.84 [0.38, 1.64]	2.01 [1.11, 2.70]	< 0.001	1.05 [0.61, 2.19]	
TPSA (µg/L)		4.83 [1.59, 12.42]	10.29 [1.18, 87.04]	0.215	5.34 [1.43, 15.34]	
FPSA (µg/L)		0.71 [0.43, 1.68]	1.44 [0.46, 4.10]	0.145	0.91 [0.45, 2.31]	
FTPSA		0.19 [0.12, 0.27]	0.11 [0.05, 0.31]	0.043	0.15 [0.09, 0.27]	
NSE (µg/L)		10.70 [8.50, 13.60]	9.80 [8.70, 13.35]	0.754	10.00 [8.50, 13.60]	
AFP (µg/L)		3.00 [2.20, 4.30]	2.90 [1.72, 4.18]	0.486	3.00 [1.90, 4.30]	
CEA (µg/L)		2.11 [1.59, 2.80]	2.32 [1.98, 2.75]	0.354	2.13 [1.88, 2.76]	
SF (µg/L)		403.00 [266.50, 558.50]	313.50 [165.00, 557.25]	0.184	372.00 [216.00, 564.00]	
β-HCG (U/L)		0.10 [0.10, 0.19]	0.10 [0.10, 0.25]	0.730	0.10 [0.10, 0.21]	
CA125 (kU/L)		13.36 [11.26, 17.65]	10.12 [7.79, 16.41]	0.051	11.88 [9.67, 17.19]	
CA15-3 (kU/L)		10.69 [8.20, 14.89]	14.27 [9.69, 15.91]	0.106	12.06 [8.65, 15.74]	
CA72-4 (kU/L)		3.93 [1.91, 8.62]	2.92 [2.07, 8.53]	0.590	3.71 [2.05, 8.84]	
CA19-9 (kU/L)		10.70 [5.65, 23.37]	10.25 [5.65, 20.80]	0.660	10.70 [5.65, 22.02]	

Data are n(%) or median [interquartile range] (for variables with skewed distribution); STK1p = serum thymidine kinase 1 protein; TPSA = total prostate-specific antigen; FPSA = free prostate-specific antigen; FTPSA = free/total prostate-specific antigen ratio; AFP = alpha-fetoprotein; CEA = carcinoembryonic antigen; SF = serum ferritin; NSE = neuron-specific enolase; β-HCG = β-human chorionic gonadotropin; CA125 = cancer antigen 125; CA15-3 = cancer antigen 15 − 3; CA72-4 = cancer antigen 72 − 4; CA19-9 = cancer antigen 19 − 9.

### Statistical analysis

Statistical analyses were performed using R version 4.2.2 and Stata MP version 17.0. All the statistical tests were two-sided, and a p value less than 0.05 was considered to indicate statistical significance. The predictive mean matching method was used for the imputation of missing candidate predictors, while k-nearest neighbors imputation with k set to five was used. The Wilcoxon rank-sum test was used to compare the levels of continuous variables (as they had skewed distributions) between the PCA and non-PCA groups. The number needed to biopsy (NNB) was calculated as the inverse of the absolute risk difference between the PCA and non-PCA groups.

## Results

### Participants

A total of 310 men met the inclusion and exclusion criteria and were included in this study. The age of the 310 study participants ranged from 32 to 93 years; 126 (40.65%) patients had positive biopsy results (PCA) (Gleason score: 6–9), and the remaining 184 non-PCA patients had negative biopsy results (moderate or severe BPH). Age (*P* = 0.001), irregular margin (*P* < 0.001), STK1p (*P* < 0.001), TPSA (*P* = 0.02), FPSA (*P* = 0.001), and FTPSA (*P* = 0.013) were significantly different between the PCA and non-PCA groups (Table 1). There were no significant differences in the other candidate predictors between the two groups. These trends were also observed in both the training and test sets (Table 1).

### Machine learning and logistic models

**Fig. 2:**
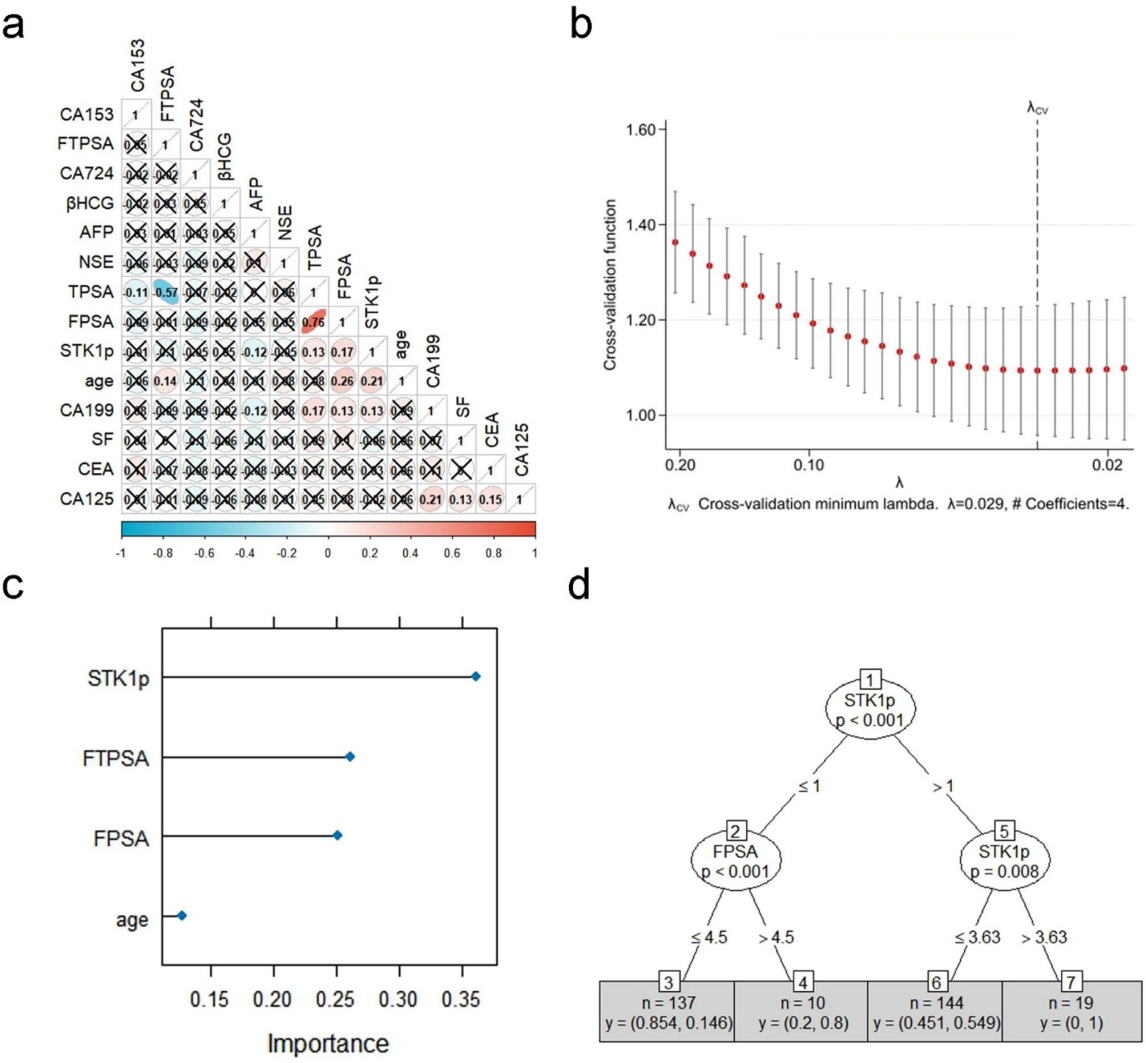
Prostatic carcinoma risk assessment model development and validation. (**a**) Spearman correlation coefficients (r) between each candidate predictor. A square with a ╳ indicates *p* ≥ 0.05; otherwise, *p* < 0.05. (**b**) Predictor selection for the final machine learning models based on the Lasso procedure. (**c**) Importance of predictors included in the XGBoost model based on Shapley values. (**d**) The visualized decision tree learning model.

The TRUS-detected irregular prostate margin was not included in the predictor selection due to the lack of positive samples (*n* = 3 in the non-PCA group) (Fig. 1b). The missing data rates of the remaining candidate predictors are presented in Table 2. The Spearman correlation coefficients between each candidate predictor are presented in Fig. 2a. No strong correlations were found between STK1p and the other candidate predictors (*r* < 0.75). In the Lasso procedure, four predictors, namely, STK1p, FPSA, age, and FTPSA, were selected for the development of machine learning models (Supplementary Table 1; Fig. 2b). Three predictors, namely, STK1p, age, and TPSA, were selected for the logistic regression model.

**Table 2:** Amount of missingness of included predictors.

Variables	Missing	Non-missing	Missing rate
Age	0	310	0.00%
STK1p	0	310	0.00%
TPSA	31	279	10.00%
FPSA	98	212	31.61%
FTPSA	98	212	31.61%
AFP	102	208	32.90%
CEA	102	208	32.90%
SF	102	208	32.90%
CA125	106	204	34.19%
CA153	106	204	34.19%
βHCG	107	203	34.52%
CA724	107	203	34.52%
NSE	107	203	34.52%
CA199	107	203	34.52%

STK1p = serum thymidine kinase 1 protein; TPSA = total prostate-specific antigen; FPSA = free prostate-specific antigen; FTPSA = free/total prostate-specific antigen ratio; AFP = alpha-fetoprotein; CEA = carcinoembryonic antigen; SF = serum ferritin; NSE = neuron-specific enolase; β-HCG = β-human chorionic gonadotropin; CA125 = cancer antigen 125; CA15-3 = cancer antigen 15 − 3; CA72-4 = cancer antigen 72 − 4; CA19-9 = cancer antigen 19 − 9.

The XGBOOST model had a greater AUC (0.965) than the other machine learning models (SVM: 0.708, decision tree learning: 0.739, NNET: 0.747, Lasso: 0.817) (*P* < 0.001), demonstrating excellent performance (Table 3). The NNB to detect one additional case of PCA using the XGBOOST model was 1.07, which was lower than other models. This metric supports the practical use of the XGBOOST model. In the XGBOOST model, the importance of predictors included in the XGBOOST model, calculated by the Shapley values, is presented in Fig. 2c. The XGBOOST model generated 49 gradient-boosted decision trees. Visualized XGBOOST decision trees are provided in supplementary Fig. 1 to help interpret the model’s decisions. The decision tree learning model generated one decision tree that included STK1p and FPSA (Fig. 2d).

**Table 3:** Performance of the machine learning models and the logistic regression model.

Model	AUC (95% confidence interval)	Cutoff	Sensitivity	Specificity	NNB
**Machine learning models**					
XGBOOST (1 vs. 0)	0.965 (0.944, 0.987)	≥ 1	95%	98%	1.07
LASSO (probability)	0.817 (0.771, 0.864)	≥ 22%	91%	52%	2.17
Neural network (1 vs. 0)	0.747 (0.698, 0.796)	≥ 1	62%	88%	1.81
Decision tree (1 vs. 0)	0.739 (0.691, 0.786)	≥ 1	84%	64%	2.13
Support vector machine (1 vs. 0)	0.708 (0.658, 0.759)	≥ 1	56%	85%	2.19
**Logistic model**					
Logistic model (probability)	0.813 (0.766, 0.861)	≥ 22%	91%	53%	2.14

AUC = area under the receiver operating characteristic curve; NNB = number needed to biopsy.

Each XGBOOST tree makes decisions based on the predictor values of an input individual and outputs a prediction via PCA (supplementary Fig. 1). The steps of the prediction are as follows:

1. Input individual: Start with a new individual whom you want to predict. The individual should have values for the predictors used in the trees (STK1p, age, FPSA, and FTPSA).
2. Traverse the tree: For each tree, start at the root node and apply the splitting rules based on the sample’s feature values.
3. Reach a leaf node: Continue traversing until you reach a leaf node. The “value” at the leaf node is the contribution of that tree to the final prediction.
4. Aggregate predictions: Since XGBoost uses an ensemble of trees, steps 2 and 3 are repeated for all trees in the model. The “value” from each tree’s leaf node is summed to obtain the aggregate prediction.
5. Final raw prediction: The sum of the contributions from all trees provides the final prediction. As this model applies a binary classification task, the raw prediction is converted into a class label.
6. Apply a threshold: The raw prediction is then compared against a threshold to determine the class label. The threshold is set to 0.5 for a binary classification task.
7. Convert to class labels: If the raw prediction is ≥ 0.5, the individual is assigned to the positive class (1 represents high PCA risk). If the raw prediction is < 0.5, the individual is assigned to the negative class (0 represents low PCA risk).

Three predictors, namely, STK1p, age, and TPSA, were selected for the forward stepwise method to develop a logistic model (Table 4). These predictors all showed a significant association with PCA in the multivariable logistic model (Table 4). The level of multicollinearity between variables included in the logistic regression model was low (Supplementary Table 2). The AUC of the logistic model was 0.813, demonstrating satisfactory performance.

**Table 4:** Univariable and multivariable logistic models for prostatic carcinoma.

Variable	Univariable			Multivariable			
	OR	95% confidence interval	*P*	OR	95% confidence interval	Coefficient	*P*
STK1p (pmol/L)	2.33	1.82, 2.97	< 0.001	2.17	1.68, 2.81	0.7762	< 0.001
Age (year)	1.05	1.02, 1.08	0.001	1.04	1.004, 1.07	0.0358	0.029
TPSA (µg/L)	1.02	1.01, 1.03	< 0.001	1.02	1.01, 1.03	0.0183	< 0.001
Baseline odds	-	-	-	0.01	0.001, 0.11	-4.5551	< 0.001

*n* = 310; OR = odds ratio; STK1p = serum thymidine kinase 1 protein; TPSA = total prostate-specific antigen.

### Model performance

**Fig. 3:**
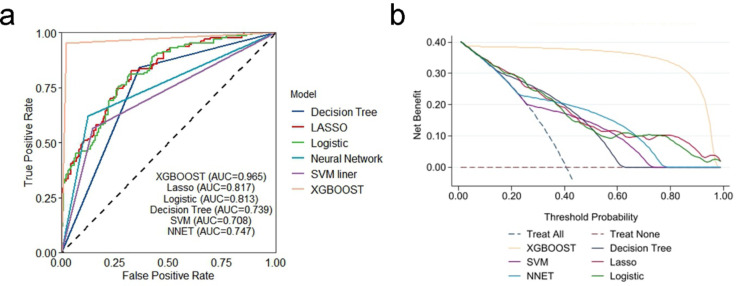
(**a**) Receiver operating characteristic (ROC) curves of each model. SVM = support vector machine model; NNET = neural network model; AUC = area under the ROC curve. (**b**) Decision curves of each model. ‘treat all’ assumes that all samples received the intervention, and the net benefit curve shows a negative slope; ‘treat none’ assumes that no samples received the intervention, and the net benefit is zero.

The AUCs of the different models are presented in Fig. 3a. The AUC of the logistic model was lower than that of the XGBOOST model (DeLong test: *P* < 0.001). The cutoff point of PCA risk (probability) assessed by the logistic regression model was 22%, and the corresponding sensitivity (91%) and specificity (53%) were 91% and 91%, respectively. The XGBOOST model performed better than the other machine learning models and logistic models in terms of sensitivity and specificity (Table 4). Decision curve analysis (Fig. 3b) revealed that the net benefit of the XGBOOST model was consistently greater than that of the other models.

Supplementary Table 2 compares the performance of the XGBoost and logistic models across different age and center subgroups in terms of their ability to assess PCA risk. In each center and each age subgroup except for the 30–60 years age group, the XGBoost model had a greater AUC than did the logistic model (*P* < 0.05), indicating better performance. In the 30- to 60-year-old age group, there was no significant difference in the AUC between the two models (*P* = 0.807).

**Fig. 4:**
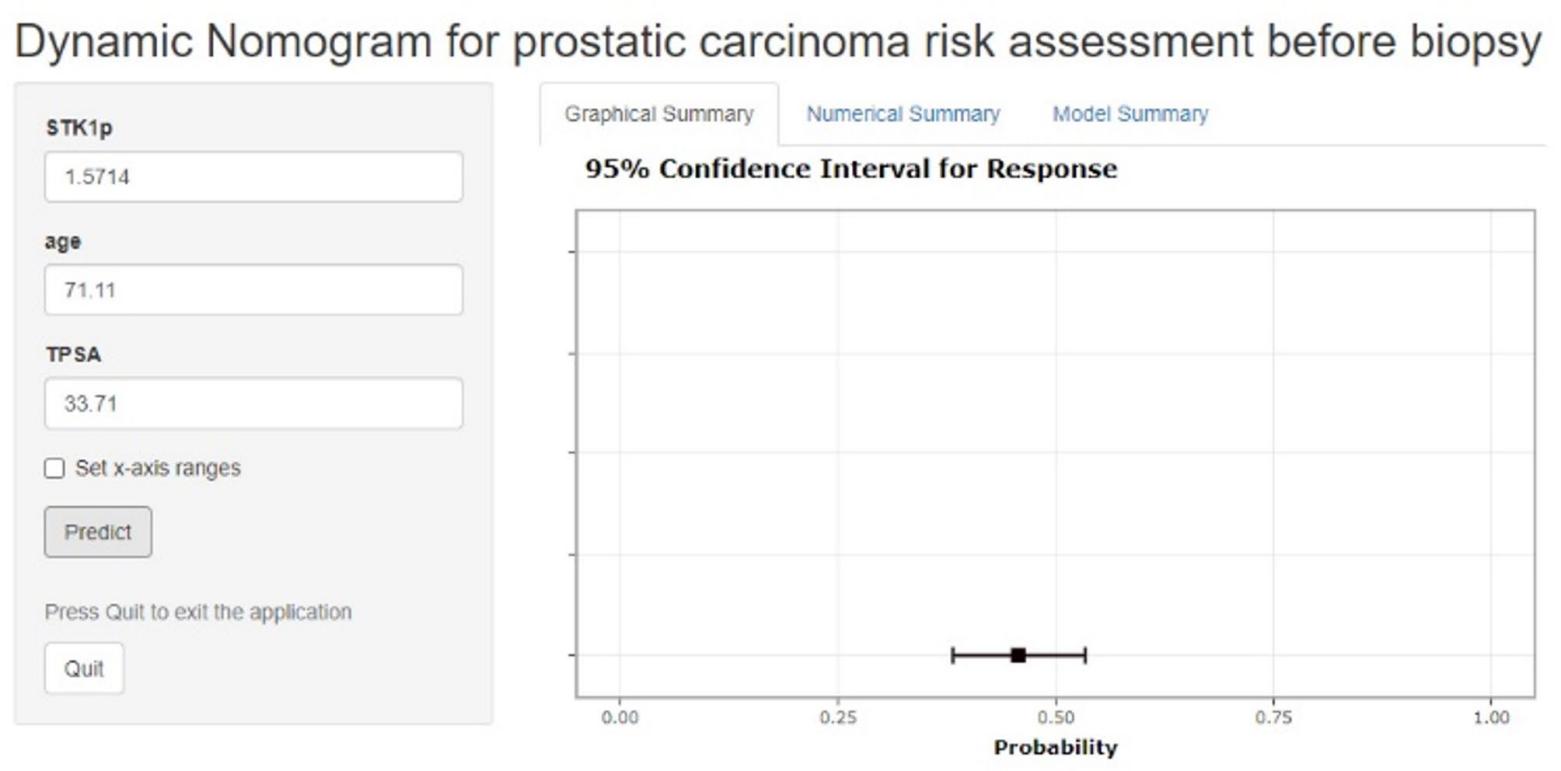
Online risk assessment tool accessible at https://pcariskprebiopsy.shinyapps.io/dynnomapp.

Due to limited funding, we were unable to build an online calculator based on the XGBOOST model. To promote clinical translation, we have developed an online calculator based on the logistic model (Fig. 4), which shows satisfactory performance. We will actively seek additional resources and collaborations to develop an online calculator based on the XGBOOST model in the future.

## Discussion

This study developed a visualized machine learning XGBOOST model for assessing PCA risk in patients with moderate or severe BPH. The model was based on noninvasive parameters, including STK1p, age, FPSA, and FTPSA. The XGBOOST model was well discriminated and had a better performance than other machine learning models and a logistic model. The XGBOOST model can help precisely select patients at high risk of PCA for subsequent biopsies.

The values of STK1p, age, FPSA, and FTPSA for PCA risk assessment were in line with previous studies. Several studies have shown that STK1p levels are greater in PCA patients than in patients with benign prostate diseases or healthy individuals^11,13–15^. Previous data on the global cancer burden indicate that the risk of prostate cancer in China increases with age^16^. Similarly, the present study showed that age was positively associated with prostate cancer risk. The FPSA has been extensively studied in relation to PCA detection^17^. Research has also shown that the combination of various parameters, including FTPSA, can significantly enhance PCA detection rates (Wang et al. 2006). The current study also reported similar associations. Our study also highlights the importance of establishing standardized protocols for the measurement and interpretation of biomarkers to ensure consistency and reliability across different populations.

Other biomarkers were found to be associated with PCA in previous studies, but not in the present study. For example, elevated CA153 levels have been found in various types of cancer, including PCA^18^, and might be used as a potential marker. NSE is an enzyme found in neuroendocrine cells. Its role in PCA has been less defined, but it has been reported as a marker in certain situations, particularly in progressive and metastatic castration-resistant PCA^19^. CEA is a tumor marker that can be elevated in various types of cancer, including advanced PCA^20^. The reasons why the present study did not find such associations might include the small sample size, different measurements, and different populations. Therefore, a larger study sample size is required to further investigate these observations.

A single predictor such as the TPSA may not be sufficient to accurately assess PCA risk^3^. Therefore, this study developed a novel multivariable model that may assist in the accurate identification of high-risk individuals to optimize PCA screening strategies. Currently, China’s PCA screening guidelines recommend the use of TPSA as a predictor for early screening, and its sensitivity is 91%^3,4^. However, the specificity of TPSA in early screening for PCA in the Chinese population is as low as 41%, which corresponds to a high rate of false positives and unnecessary biopsies^4^. This study used a novel machine learning algorithm, XGBOOST, which is highly efficient, flexible, and explanatory in previous model development studies^21^. The XGBOOST model performed better than the traditional logistic model in PCA risk assessment in the current study. The XGBOOST model had both high sensitivity (95%) and specificity (98%) and may serve as a robust, accurate, and interpretable tool that can potentially improve clinical outcomes by facilitating the precise detection of PCA risk. Moreover, the XGBOOST decision trees were visually presented to explain the model’s decisions, which is crucial for healthcare workers to understand the model in clinical practice.

In the error analysis of the XGBOOST model, we identified two primary types of errors: false positives (2%), where the model incorrectly diagnosed BPH as PCA, and false negatives (5%), where PCA was misclassified as BPH. Upon reviewing the cases of misclassification, we observed that false positives tended to occur in patients with elevated levels of TPSA but without the presence of other discriminating factors such as STK1p. False negatives were noted in cases where PCA presented with atypical biomarker profiles. The feature importance indicated that STK1p, FTPSA, and FPSA were the most influential in predictions, aligning with current medical understanding. To address these errors, we plan to refine our model by adjusting the decision threshold, exploring alternative feature engineering techniques, and increasing the diversity of our training dataset to better capture the variability in PCA and BPH presentations. Future work will focus on external validation of the model with additional datasets to further assess and improve its predictive accuracy.

This study has several limitations. The small number of samples and centers included in this study might have led to a lack of representativeness, for which we will further expand the study sample size. In addition, the small sample size could not allow the performance of subgroup analysis based on the Gleason score or other variables. We will further analyze the performance of the model in different subgroups in our subsequent research to improve PCA risk assessment. Due to the lack of data on PCA risk factors such as ethnicity, family history, and lifestyle factors in routine healthcare settings, the feasibility of including these factors in the model could not be assessed in this study. We will adopt a questionnaire to improve the collection of these data and explore whether these indicators can further improve the performance of the model. Our study did not include data on digital rectal examination (DRE), prostate-specific antigen (PSA) density, and magnetic resonance imaging (MRI), which may play an important role in improving the performance of a model for PCA^22^. However, these measurements were not routinely used for PCA detection in the study centers, which may have affected the comprehensiveness of our results. A further limitation of our study is that all participants were recruited from two centers in China. The distribution of biomarkers such as STK1p and PSA may differ in Western or other Asian populations, which could limit the generalizability of our findings. We encourage future studies to conduct external validation of our findings in multiethnic, international cohorts to assess the generalizability of our results across different populations. Moreover, due to the limited sample size, we were unable to perform analyses separating Gleason ≥ 7 vs. Gleason 6 or clinically significant PCA (csPCA) vs. non-csPCA. Future studies with larger sample sizes are needed to explore these clinically meaningful distinctions.

## Conclusion

In conclusion, this study developed a novel visualized XGBOOST model based on eight noninvasive parameters, such as STK1p and FTPSA, which was accurate for assessing PCA risk in patients with moderate or severe prostatic hyperplasia. Identifying high-risk patients based on the model may be beneficial for optimizing the identification of patients for biopsy, ensuring timely PCA diagnosis, and reducing complications and costs due to unnecessary biopsies. However, big data and multicenter studies are still needed to further validate the efficacy of the model, and more PCA risk assessment indicators should be investigated to further improve the performance of the model.

## Supplementary Information

Below is the link to the electronic supplementary material.

Supplementary Material 1

## Data Availability

The data that support the findings of this study are not openly available due to reasons of sensitivity and are available from the corresponding author upon reasonable request.
