# Supplementary material for: A visualized machine learning model using noninvasive parameters to differentiate men with and without prostatic carcinoma before biopsy
Source: Sci Rep. 2025 Jul 27;15:27357. doi: 10.1038/s41598-025-12765-2 (PMC12301460; doi:10.1038/s41598-025-12765-2)
Supplement: Supplementary file 1 — Supplementary Material 1 [file 41598_2025_12765_MOESM1_ESM.pdf]

Supplementary table 1. Final predictors for developing machine learning models selected by the Lasso procedure.

Lambda	No. of non-zero coefficient	Cross-validation mean deviance	Variables (A)dded, (R)emoved, or left (U)nchanged	
0.1874	1	1.3399	A	STK1p
0.1177	2	1.2365	A	FPSA
0.0673	3	1.1507	A	age
0.0423	4	1.1182	A	FTPSA
0.0291*	4	1.1076	U	

* Lambda selected by the minimum cross-validation mean deviance. STK1p = serum thymidine kinase 1 protein; FPSA = free prostate-specific antigen; FTPSA = free/total prostate-specific antigen ratio.

Supplementary table 2. Multicollinearity test following logistic model

Variable	Variance inflation factor	Tolerance
STK1p (pmol/L)	1.01	0.99
Age (year)	1.01	0.99
TPSA (μg/L)	1.00	1.00

STK1p=serum thymidine kinase 1 protein; TPSA=total prostate-specific antigen

Supplementary table 3. Performance comparison between the XGBOOST and logistic model by subgroups.

Subgroups	n	Model	AUC (95% confidence interval)	P
Overall	310	XGBOOST	0.965 (0.944, 0.987)	**<0.001**
		Logistic	0.813 (0.766, 0.861)	
Aged 30-60 years	29	XGBOOST	0.938 (0.815, 1.000)	0.807
		Logistic	0.929 (0.822, 1.000)	
Aged 60-69 years	93	XGBOOST	0.960 (0.913, 1.000)	**0.031**
		Logistic	0.858 (0.772, 0.944)	
Aged 70-79 years	137	XGBOOST	0.962 (0.928, 0.995)	**<0.001**
		Logistic	0.738 (0.655, 0.821)	
Aged≥80 years	51	XGBOOST	0.978 (0.936, 1.000)	**0.001**
		Logistic	0.784 (0.659, 0.909)	
Center 1	212	XGBOOST	0.931 (0.886, 0.976)	**<0.001**
		Logistic	0.727 (0.649, 0.805)	
Center 2	98	XGBOOST	1.000 (1.000, 1.000)	**0.022**
		Logistic	0.938 (0.885, 0.991)	

AUC = area under the receiver operating characteristic curve; P values were provided by the DeLong test for comparing AUCs. Center 1 refers to the First People's Hospital of Longquanyi District; center 2 refers to the Daping Hospital of the Third Military Medical University.

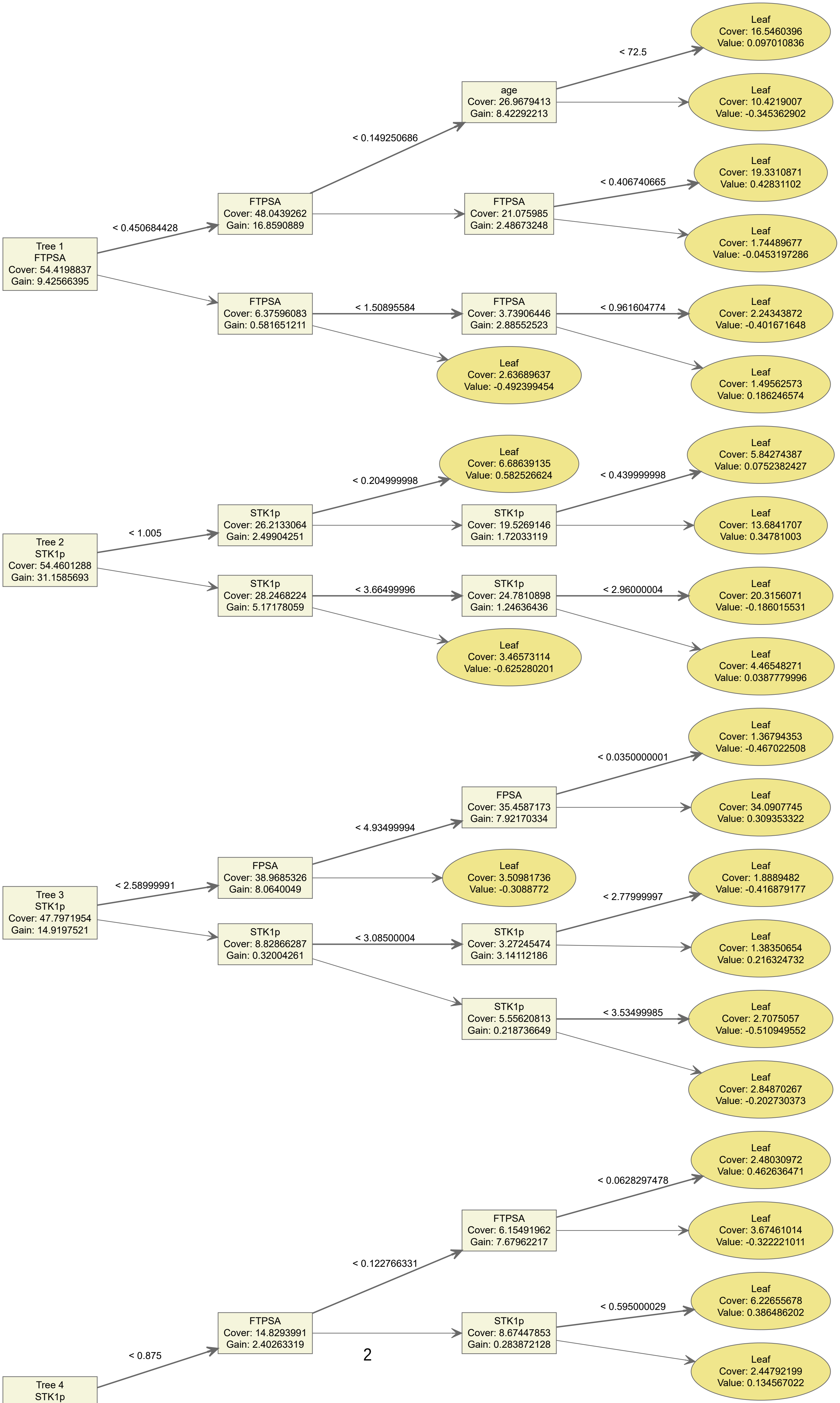
Tree 1
FTPSA
Cover: 54.4198837
Gain: 9.42566395
< 0.450684428
FTPSA
Cover: 48.0439262
Gain: 16.8590889
< 0.149250686
age
Cover: 26.9679413
Gain: 8.42292213
< 72.5
Leaf
Cover: 16.5460396
Value: 0.097010836
Leaf
Cover: 10.4219007
Value: -0.345362902
FTPSA
Cover: 21.075985
Gain: 2.48673248
< 0.406740665
Leaf
Cover: 19.3310871
Value: 0.42831102
Leaf
Cover: 1.74489677
Value: -0.0453197286
FTPSA
Cover: 6.37596083
Gain: 0.581651211
< 1.50895584
FTPSA
Cover: 3.73906446
Gain: 2.88552523
< 0.961604774
Leaf
Cover: 2.24343872
Value: -0.401671648
Leaf
Cover: 1.49562573
Value: 0.186246574
Leaf
Cover: 2.63689637
Value: -0.492399454
Tree 2
STK1p
Cover: 54.4601288
Gain: 31.1585693
< 1.005
STK1p
Cover: 26.2133064
Gain: 2.49904251
< 0.204999998
Leaf
Cover: 6.68639135
Value: 0.582526624
STK1p
Cover: 19.5269146
Gain: 1.72033119
< 0.439999998
Leaf
Cover: 5.84274387
Value: 0.0752382427
Leaf
Cover: 13.6841707
Value: 0.34781003
STK1p
Cover: 28.2468224
Gain: 5.17178059
< 3.66499996
STK1p
Cover: 24.7810898
Gain: 1.24636436
< 2.96000004
Leaf
Cover: 20.3156071
Value: -0.186015531
Leaf
Cover: 4.46548271
Value: 0.0387779996
Leaf
Cover: 3.46573114
Value: -0.625280201
Tree 3
STK1p
Cover: 47.7971954
Gain: 14.9197521
< 2.58999991
FPSA
Cover: 38.9685326
Gain: 8.0640049
< 4.93499994
FPSA
Cover: 35.4587173
Gain: 7.92170334
< 0.0350000001
Leaf
Cover: 1.36794353
Value: -0.467022508
Leaf
Cover: 34.0907745
Value: 0.309353322
Leaf
Cover: 3.50981736
Value: -0.3088772
STK1p
Cover: 8.82866287
Gain: 0.32004261
< 3.08500004
STK1p
Cover: 3.27245474
Gain: 3.14112186
< 2.77999997
Leaf
Cover: 1.8889482
Value: -0.416879177
Leaf
Cover: 1.38350654
Value: 0.216324732
STK1p
Cover: 5.55620813
Gain: 0.218736649
< 3.53499985
Leaf
Cover: 2.7075057
Value: -0.510949552
Leaf
Cover: 2.84870267
Value: -0.202730373
Tree 4
STK1p
< 0.875
FTPSA
Cover: 14.8293991
Gain: 2.40263319
< 0.122766331
FTPSA
Cover: 6.15491962
Gain: 7.67962217
< 0.0628297478
Leaf
Cover: 2.48030972
Value: 0.462636471
Leaf
Cover: 3.67461014
Value: -0.322221011
STK1p
Cover: 8.67447853
Gain: 0.283872128
< 0.595000029
Leaf
Cover: 6.22655678
Value: 0.386486202
Leaf
Cover: 2.44792199
Value: 0.134567022

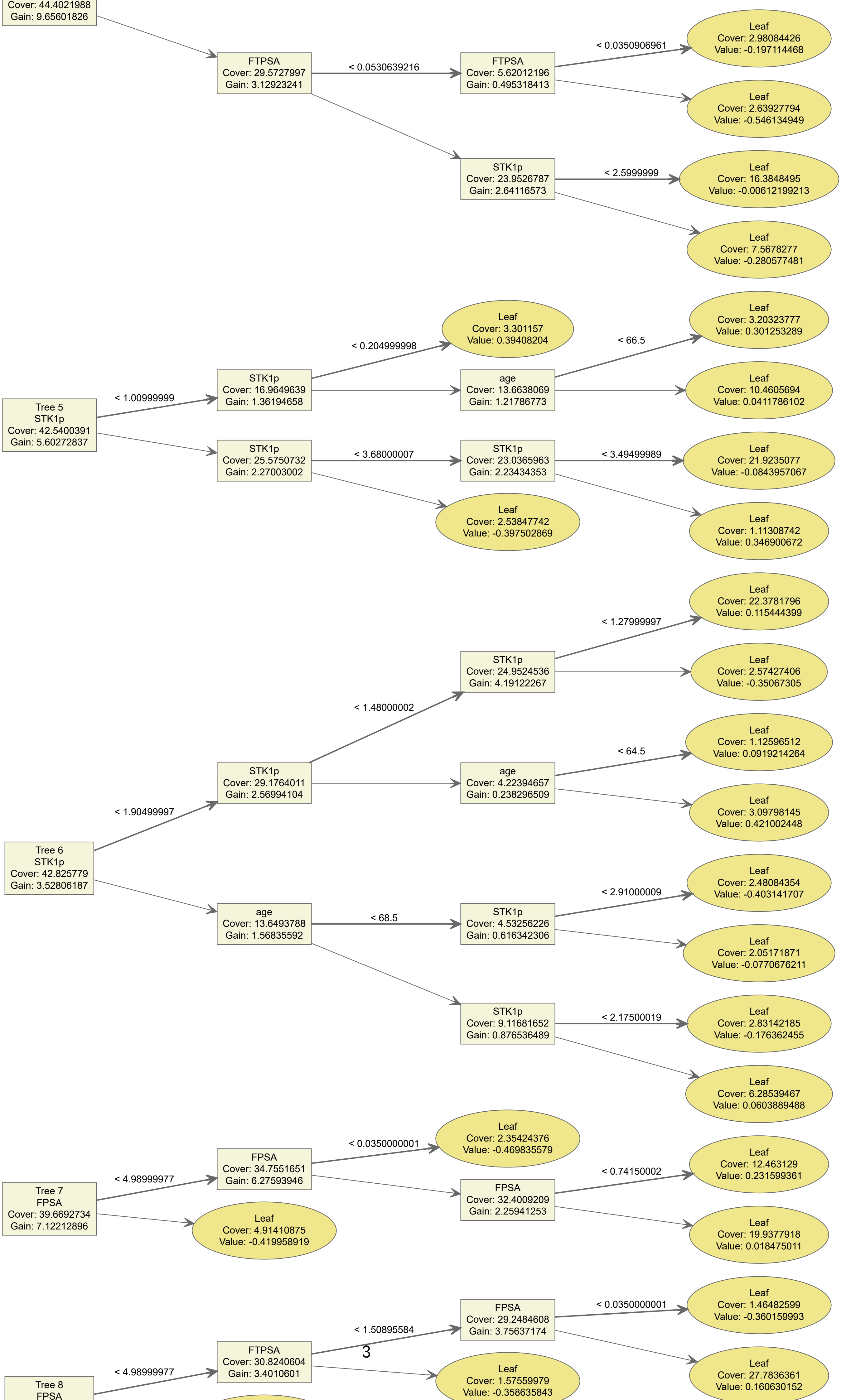
Cover: 44.4021988
Gain: 9.65601826
FTPSA
Cover: 29.5727997
Gain: 3.12923241
< 0.0530639216
FTPSA
Cover: 5.62012196
Gain: 0.495318413
< 0.0350906961
Leaf
Cover: 2.98084426
Value: -0.197114468
Leaf
Cover: 2.63927794
Value: -0.546134949
STK1p
Cover: 23.9526787
Gain: 2.64116573
< 2.5999999
Leaf
Cover: 16.3848495
Value: -0.00612199213
Leaf
Cover: 7.5678277
Value: -0.280577481
Tree 5
STK1p
Cover: 42.5400391
Gain: 5.60272837
< 1.00999999
STK1p
Cover: 16.9649639
Gain: 1.36194658
< 0.204999998
Leaf
Cover: 3.301157
Value: 0.39408204
age
Cover: 13.6638069
Gain: 1.21786773
< 66.5
Leaf
Cover: 3.20323777
Value: 0.301253289
Leaf
Cover: 10.4605694
Value: 0.0411786102
STK1p
Cover: 25.5750732
Gain: 2.27003002
< 3.68000007
STK1p
Cover: 23.0365963
Gain: 2.23434353
< 3.49499989
Leaf
Cover: 21.9235077
Value: -0.0843957067
Leaf
Cover: 1.11308742
Value: 0.346900672
Leaf
Cover: 2.53847742
Value: -0.397502869
Tree 6
STK1p
Cover: 42.825779
Gain: 3.52806187
< 1.90499997
STK1p
Cover: 29.1764011
Gain: 2.56994104
< 1.48000002
STK1p
Cover: 24.9524536
Gain: 4.19122267
< 1.27999997
Leaf
Cover: 22.3781796
Value: 0.115444399
Leaf
Cover: 2.57427406
Value: -0.35067305
age
Cover: 4.22394657
Gain: 0.238296509
< 64.5
Leaf
Cover: 1.12596512
Value: 0.0919214264
Leaf
Cover: 3.09798145
Value: 0.421002448
age
Cover: 13.6493788
Gain: 1.56835592
< 68.5
STK1p
Cover: 4.53256226
Gain: 0.616342306
< 2.91000009
Leaf
Cover: 2.48084354
Value: -0.403141707
Leaf
Cover: 2.05171871
Value: -0.0770676211
STK1p
Cover: 9.11681652
Gain: 0.876536489
< 2.17500019
Leaf
Cover: 2.83142185
Value: -0.176362455
Leaf
Cover: 6.28539467
Value: 0.0603889488
Tree 7
FPSA
Cover: 39.6692734
Gain: 7.12212896
< 4.98999977
FPSA
Cover: 34.7551651
Gain: 6.27593946
< 0.0350000001
Leaf
Cover: 2.35424376
Value: -0.469835579
FPSA
Cover: 32.4009209
Gain: 2.25941253
< 0.74150002
Leaf
Cover: 12.463129
Value: 0.231599361
Leaf
Cover: 19.9377918
Value: 0.018475011
Leaf
Cover: 4.91410875
Value: -0.419958919
Tree 8
FPSA
< 4.98999977
FTPSA
Cover: 30.8240604
Gain: 3.4010601
< 1.50895584
FPSA
Cover: 29.2484608
Gain: 3.75637174
< 0.0350000001
Leaf
Cover: 1.46482599
Value: -0.360159993
Leaf
Cover: 27.7836361
Value: 0.160630152
Leaf
Cover: 1.57559979
Value: -0.358635843

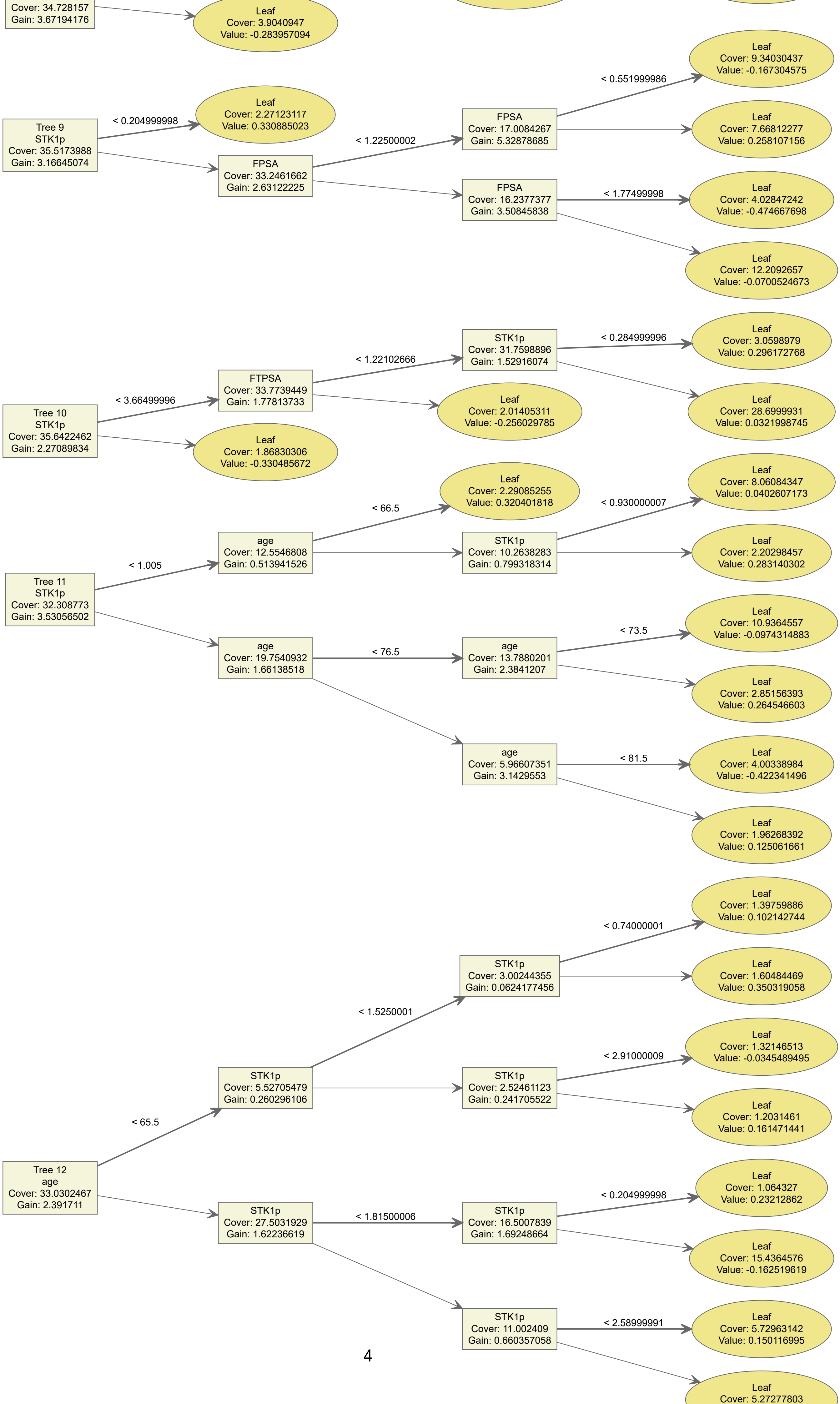

Cover: 34.728157
Gain: 3.67194176
Leaf
Cover: 3.9040947
Value: -0.283957094
Tree 9
STK1p
Cover: 35.5173988
Gain: 3.16645074
< 0.204999998
Leaf
Cover: 2.27123117
Value: 0.330885023
FPSA
Cover: 33.2461662
Gain: 2.63122225
< 1.22500002
FPSA
Cover: 17.0084267
Gain: 5.32878685
< 0.551999986
Leaf
Cover: 9.34030437
Value: -0.167304575
Leaf
Cover: 7.66812277
Value: 0.258107156
FPSA
Cover: 16.2377377
Gain: 3.50845838
< 1.77499998
Leaf
Cover: 4.02847242
Value: -0.474667698
Leaf
Cover: 12.2092657
Value: -0.0700524673
Tree 10
STK1p
Cover: 35.6422462
Gain: 2.27089834
< 3.66499996
FTPSA
Cover: 33.7739449
Gain: 1.77813733
< 1.22102666
STK1p
Cover: 31.7598896
Gain: 1.52916074
< 0.284999996
Leaf
Cover: 3.0598979
Value: 0.296172768
Leaf
Cover: 28.6999931
Value: 0.0321998745
Leaf
Cover: 2.01405311
Value: -0.256029785
Leaf
Cover: 1.86830306
Value: -0.330485672
Tree 11
STK1p
Cover: 32.308773
Gain: 3.53056502
< 1.005
age
Cover: 12.5546808
Gain: 0.513941526
< 66.5
Leaf
Cover: 2.29085255
Value: 0.320401818
STK1p
Cover: 10.2638283
Gain: 0.799318314
< 0.930000007
Leaf
Cover: 8.06084347
Value: 0.0402607173
Leaf
Cover: 2.20298457
Value: 0.283140302
age
Cover: 19.7540932
Gain: 1.66138518
< 76.5
age
Cover: 13.7880201
Gain: 2.3841207
< 73.5
Leaf
Cover: 10.9364557
Value: -0.0974314883
Leaf
Cover: 2.85156393
Value: 0.264546603
age
Cover: 5.96607351
Gain: 3.1429553
< 81.5
Leaf
Cover: 4.00338984
Value: -0.422341496
Leaf
Cover: 1.96268392
Value: 0.125061661
Tree 12
age
Cover: 33.0302467
Gain: 2.391711
< 65.5
STK1p
Cover: 5.52705479
Gain: 0.260296106
< 1.5250001
STK1p
Cover: 3.00244355
Gain: 0.0624177456
< 0.74000001
Leaf
Cover: 1.39759886
Value: 0.102142744
Leaf
Cover: 1.60484469
Value: 0.350319058
STK1p
Cover: 2.52461123
Gain: 0.241705522
< 2.91000009
Leaf
Cover: 1.32146513
Value: -0.0345489495
Leaf
Cover: 1.2031461
Value: 0.161471441
STK1p
Cover: 27.5031929
Gain: 1.62236619
< 1.81500006
STK1p
Cover: 16.5007839
Gain: 1.69248664
< 0.204999998
Leaf
Cover: 1.064327
Value: 0.23212862
Leaf
Cover: 15.4364576
Value: -0.162519619
STK1p
Cover: 11.002409
Gain: 0.660357058
< 2.58999991
Leaf
Cover: 5.72963142
Value: 0.150116995
Leaf
Cover: 5.27277803

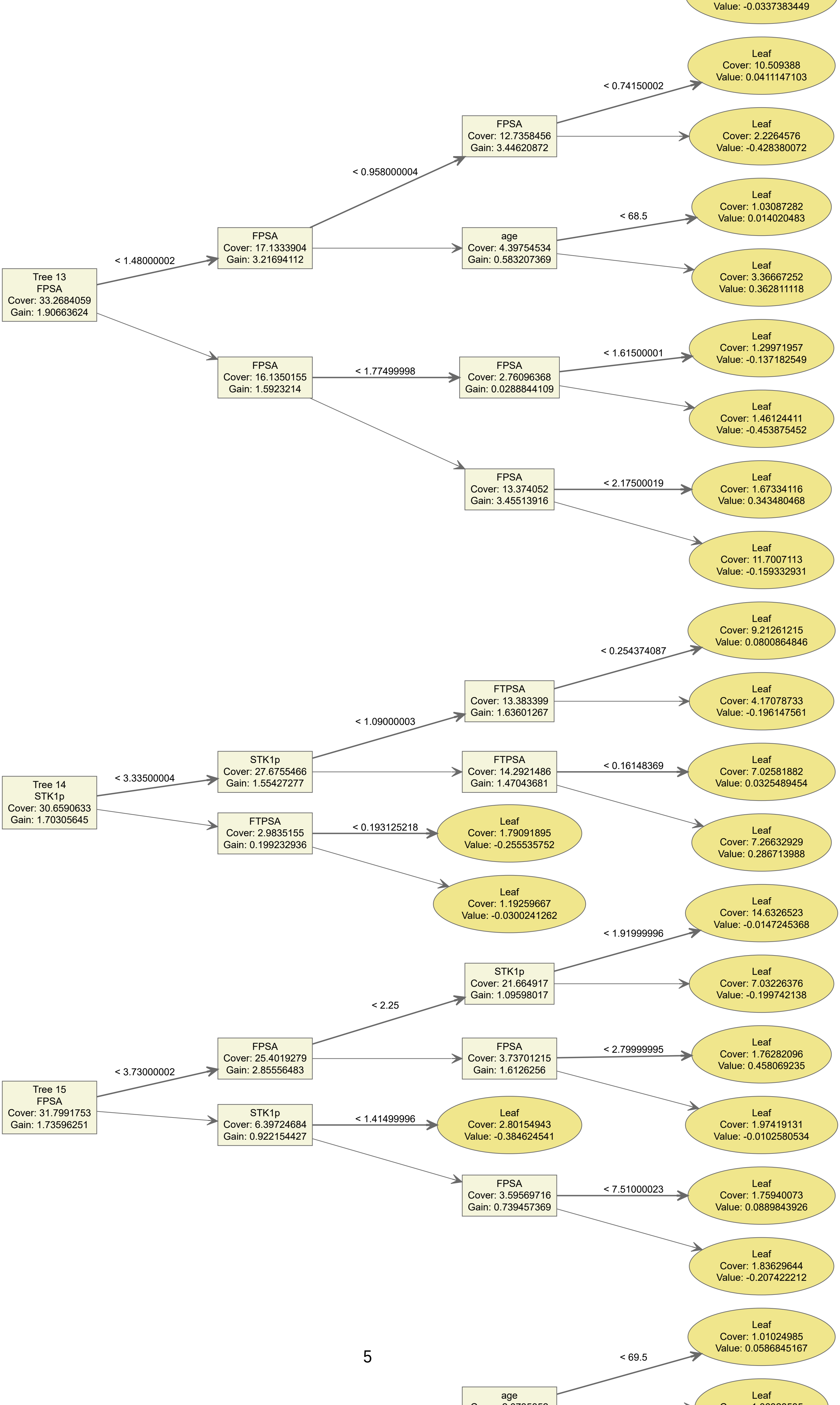
Value: -0.0337383449
Tree 13
FPSA
Cover: 33.2684059
Gain: 1.90663624
< 1.48000002
FPSA
Cover: 17.1333904
Gain: 3.21694112
< 0.958000004
FPSA
Cover: 12.7358456
Gain: 3.44620872
< 0.74150002
Leaf
Cover: 10.509388
Value: 0.0411147103
Leaf
Cover: 2.2264576
Value: -0.428380072
age
Cover: 4.39754534
Gain: 0.583207369
< 68.5
Leaf
Cover: 1.03087282
Value: 0.014020483
Leaf
Cover: 3.36667252
Value: 0.362811118
FPSA
Cover: 16.1350155
Gain: 1.5923214
< 1.77499998
FPSA
Cover: 2.76096368
Gain: 0.0288844109
< 1.61500001
Leaf
Cover: 1.29971957
Value: -0.137182549
Leaf
Cover: 1.46124411
Value: -0.453875452
FPSA
Cover: 13.374052
Gain: 3.45513916
< 2.17500019
Leaf
Cover: 1.67334116
Value: 0.343480468
Leaf
Cover: 11.7007113
Value: -0.159332931
Tree 14
STK1p
Cover: 30.6590633
Gain: 1.70305645
< 3.33500004
STK1p
Cover: 27.6755466
Gain: 1.55427277
< 1.09000003
FTPSA
Cover: 13.383399
Gain: 1.63601267
< 0.254374087
Leaf
Cover: 9.21261215
Value: 0.0800864846
Leaf
Cover: 4.17078733
Value: -0.196147561
FTPSA
Cover: 14.2921486
Gain: 1.47043681
< 0.16148369
Leaf
Cover: 7.02581882
Value: 0.0325489454
Leaf
Cover: 7.26632929
Value: 0.286713988
FTPSA
Cover: 2.9835155
Gain: 0.199232936
< 0.193125218
Leaf
Cover: 1.79091895
Value: -0.255535752
Leaf
Cover: 1.19259667
Value: -0.0300241262
Tree 15
FPSA
Cover: 31.7991753
Gain: 1.73596251
< 3.73000002
FPSA
Cover: 25.4019279
Gain: 2.85556483
< 2.25
STK1p
Cover: 21.664917
Gain: 1.09598017
< 1.91999996
Leaf
Cover: 14.6326523
Value: -0.0147245368
Leaf
Cover: 7.03226376
Value: -0.199742138
FPSA
Cover: 3.73701215
Gain: 1.6126256
< 2.79999995
Leaf
Cover: 1.76282096
Value: 0.458069235
Leaf
Cover: 1.97419131
Value: -0.0102580534
STK1p
Cover: 6.39724684
Gain: 0.922154427
< 1.41499996
Leaf
Cover: 2.80154943
Value: -0.384624541
FPSA
Cover: 3.59569716
Gain: 0.739457369
< 7.51000023
Leaf
Cover: 1.75940073
Value: 0.0889843926
Leaf
Cover: 1.83629644
Value: -0.207422212
< 69.5
Leaf
Cover: 1.01024985
Value: 0.0586845167
age
Leaf

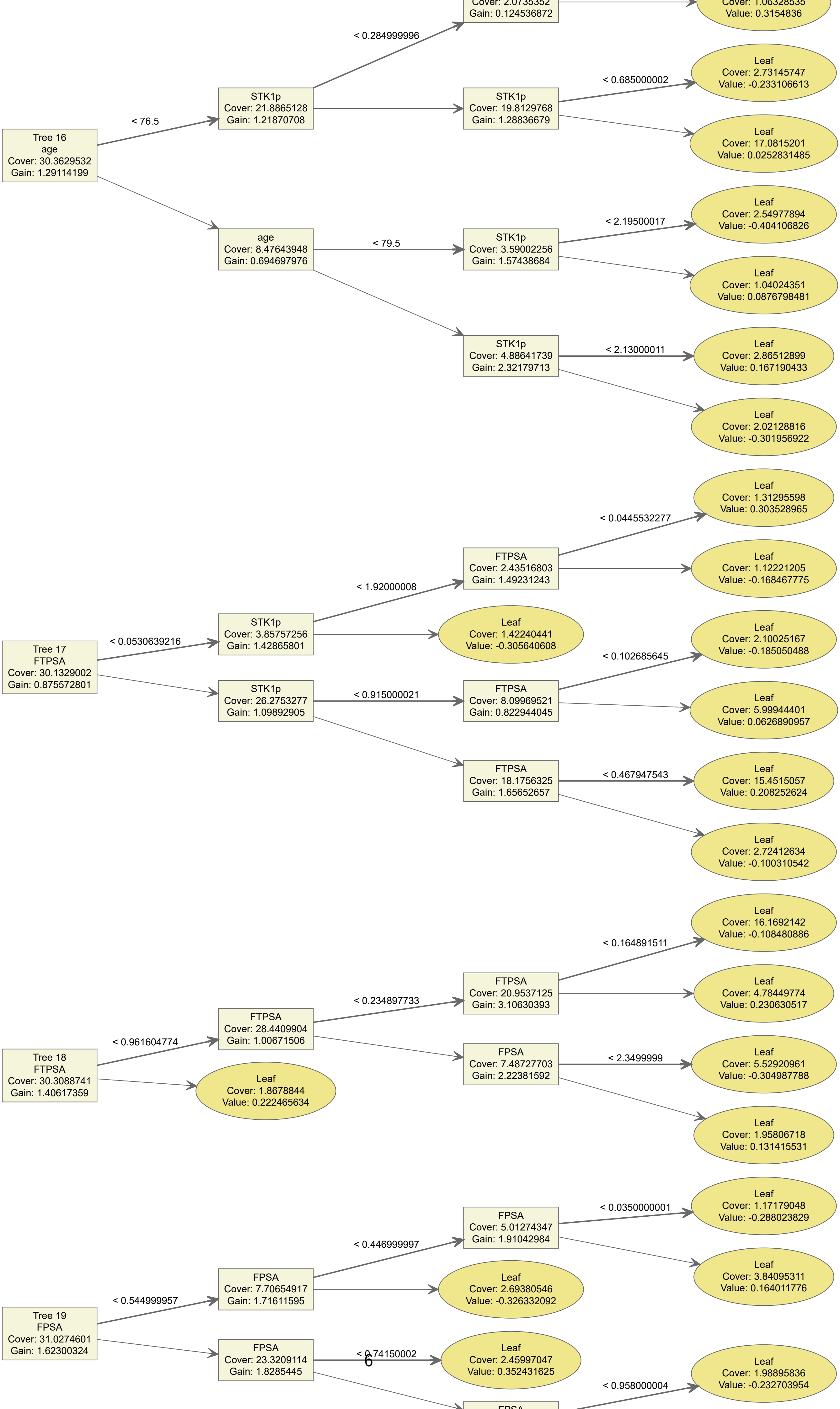

Cover: 2.0735352
Gain: 0.124536872
Cover: 1.06328535
Value: 0.3154836
< 0.284999996
Tree 16
age
Cover: 30.3629532
Gain: 1.29114199
< 76.5
STK1p
Cover: 21.8865128
Gain: 1.21870708
STK1p
Cover: 19.8129768
Gain: 1.28836679
< 0.685000002
Leaf
Cover: 2.73145747
Value: -0.233106613
Leaf
Cover: 17.0815201
Value: 0.0252831485
age
Cover: 8.47643948
Gain: 0.694697976
< 79.5
STK1p
Cover: 3.59002256
Gain: 1.57438684
< 2.19500017
Leaf
Cover: 2.54977894
Value: -0.404106826
Leaf
Cover: 1.04024351
Value: 0.0876798481
STK1p
Cover: 4.88641739
Gain: 2.32179713
< 2.13000011
Leaf
Cover: 2.86512899
Value: 0.167190433
Leaf
Cover: 2.02128816
Value: -0.301956922
Tree 17
FTPSA
Cover: 30.1329002
Gain: 0.875572801
< 0.0530639216
STK1p
Cover: 3.85757256
Gain: 1.42865801
< 1.92000008
FTPSA
Cover: 2.43516803
Gain: 1.49231243
< 0.0445532277
Leaf
Cover: 1.31295598
Value: 0.303528965
Leaf
Cover: 1.12221205
Value: -0.168467775
Leaf
Cover: 1.42240441
Value: -0.305640608
STK1p
Cover: 26.2753277
Gain: 1.09892905
< 0.915000021
FTPSA
Cover: 8.09969521
Gain: 0.822944045
< 0.102685645
Leaf
Cover: 2.10025167
Value: -0.185050488
Leaf
Cover: 5.99944401
Value: 0.0626890957
FTPSA
Cover: 18.1756325
Gain: 1.65652657
< 0.467947543
Leaf
Cover: 15.4515057
Value: 0.208252624
Leaf
Cover: 2.72412634
Value: -0.100310542
Tree 18
FTPSA
Cover: 30.3088741
Gain: 1.40617359
< 0.961604774
FTPSA
Cover: 28.4409904
Gain: 1.00671506
< 0.234897733
FTPSA
Cover: 20.9537125
Gain: 3.10630393
< 0.164891511
Leaf
Cover: 16.1692142
Value: -0.108480886
Leaf
Cover: 4.78449774
Value: 0.230630517
FPSA
Cover: 7.48727703
Gain: 2.22381592
< 2.3499999
Leaf
Cover: 5.52920961
Value: -0.304987788
Leaf
Cover: 1.95806718
Value: 0.131415531
Leaf
Cover: 1.8678844
Value: 0.222465634
Tree 19
FPSA
Cover: 31.0274601
Gain: 1.62300324
< 0.544999957
FPSA
Cover: 7.70654917
Gain: 1.71611595
< 0.446999997
FPSA
Cover: 5.01274347
Gain: 1.91042984
< 0.0350000001
Leaf
Cover: 1.17179048
Value: -0.288023829
Leaf
Cover: 3.84095311
Value: 0.164011776
Leaf
Cover: 2.69380546
Value: -0.326332092
FPSA
Cover: 23.3209114
Gain: 1.8285445
< 0.74150002
Leaf
Cover: 2.45997047
Value: 0.352431625
< 0.958000004
Leaf
Cover: 1.98895836
Value: -0.232703954
FPSA

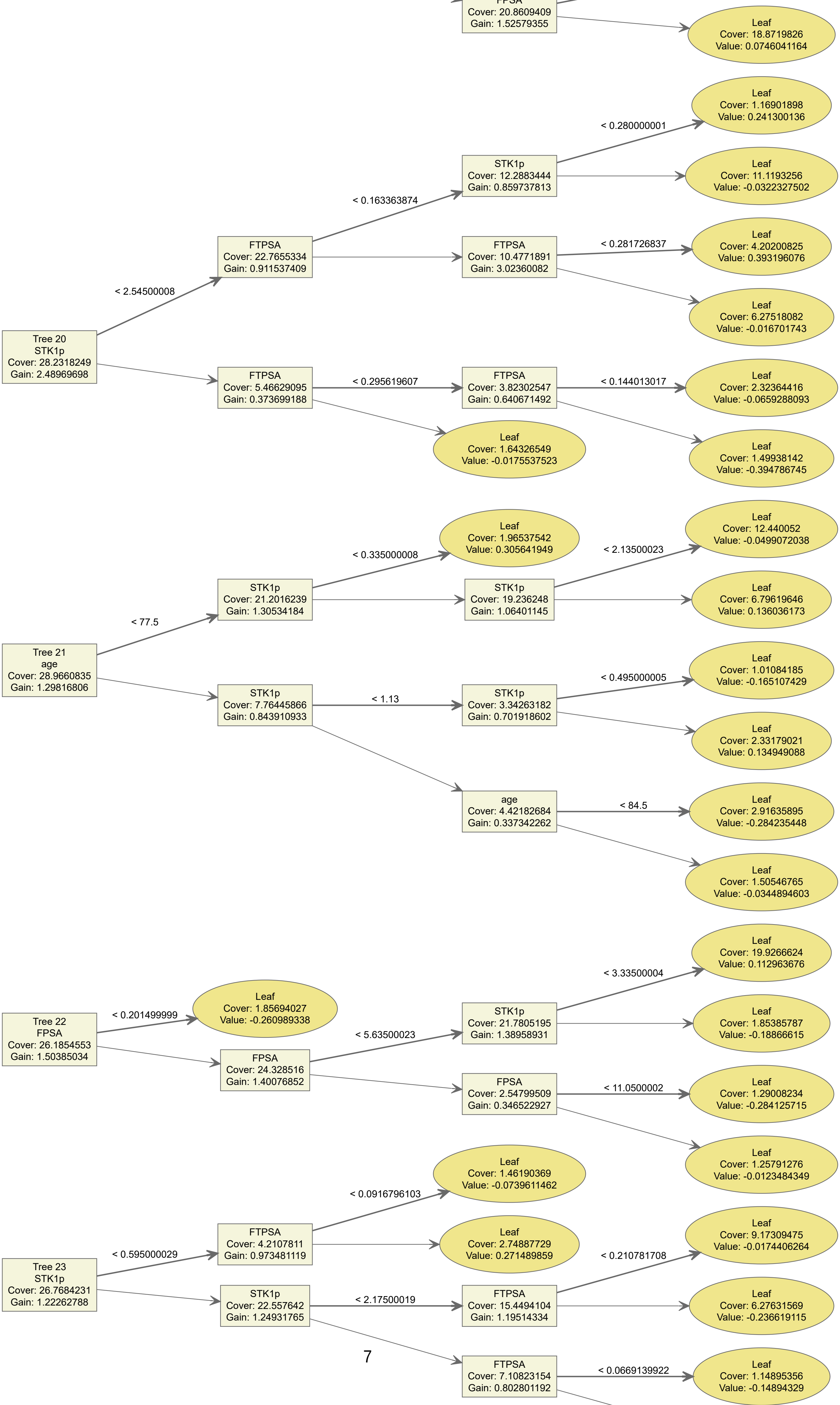
FPSA
Cover: 20.8609409
Gain: 1.52579355
Leaf
Cover: 18.8719826
Value: 0.0746041164
Tree 20
STK1p
Cover: 28.2318249
Gain: 2.48969698
< 2.54500008
FTPSA
Cover: 22.7655334
Gain: 0.911537409
< 0.163363874
STK1p
Cover: 12.2883444
Gain: 0.859737813
< 0.280000001
Leaf
Cover: 1.16901898
Value: 0.241300136
Leaf
Cover: 11.1193256
Value: -0.0322327502
FTPSA
Cover: 10.4771891
Gain: 3.02360082
< 0.281726837
Leaf
Cover: 4.20200825
Value: 0.393196076
Leaf
Cover: 6.27518082
Value: -0.016701743
FTPSA
Cover: 5.46629095
Gain: 0.373699188
< 0.295619607
FTPSA
Cover: 3.82302547
Gain: 0.640671492
< 0.144013017
Leaf
Cover: 2.32364416
Value: -0.0659288093
Leaf
Cover: 1.49938142
Value: -0.394786745
Leaf
Cover: 1.64326549
Value: -0.0175537523
Tree 21
age
Cover: 28.9660835
Gain: 1.29816806
< 77.5
STK1p
Cover: 21.2016239
Gain: 1.30534184
< 0.335000008
Leaf
Cover: 1.96537542
Value: 0.305641949
STK1p
Cover: 19.236248
Gain: 1.06401145
< 2.13500023
Leaf
Cover: 12.440052
Value: -0.0499072038
Leaf
Cover: 6.79619646
Value: 0.136036173
STK1p
Cover: 7.76445866
Gain: 0.843910933
< 1.13
STK1p
Cover: 3.34263182
Gain: 0.701918602
< 0.495000005
Leaf
Cover: 1.01084185
Value: -0.165107429
Leaf
Cover: 2.33179021
Value: 0.134949088
age
Cover: 4.42182684
Gain: 0.337342262
< 84.5
Leaf
Cover: 2.91635895
Value: -0.284235448
Leaf
Cover: 1.50546765
Value: -0.0344894603
Tree 22
FPSA
Cover: 26.1854553
Gain: 1.50385034
< 0.201499999
Leaf
Cover: 1.85694027
Value: -0.260989338
FPSA
Cover: 24.328516
Gain: 1.40076852
< 5.63500023
STK1p
Cover: 21.7805195
Gain: 1.38958931
< 3.33500004
Leaf
Cover: 19.9266624
Value: 0.112963676
Leaf
Cover: 1.85385787
Value: -0.18866615
FPSA
Cover: 2.54799509
Gain: 0.346522927
< 11.0500002
Leaf
Cover: 1.29008234
Value: -0.284125715
Leaf
Cover: 1.25791276
Value: -0.0123484349
Tree 23
STK1p
Cover: 26.7684231
Gain: 1.22262788
< 0.595000029
FTPSA
Cover: 4.2107811
Gain: 0.973481119
< 0.0916796103
Leaf
Cover: 1.46190369
Value: -0.0739611462
Leaf
Cover: 2.74887729
Value: 0.271489859
STK1p
Cover: 22.557642
Gain: 1.24931765
< 2.17500019
FTPSA
Cover: 15.4494104
Gain: 1.19514334
< 0.210781708
Leaf
Cover: 9.17309475
Value: -0.0174406264
Leaf
Cover: 6.27631569
Value: -0.236619115
FTPSA
Cover: 7.10823154
Gain: 0.802801192
< 0.0669139922
Leaf
Cover: 1.14895356
Value: -0.14894329

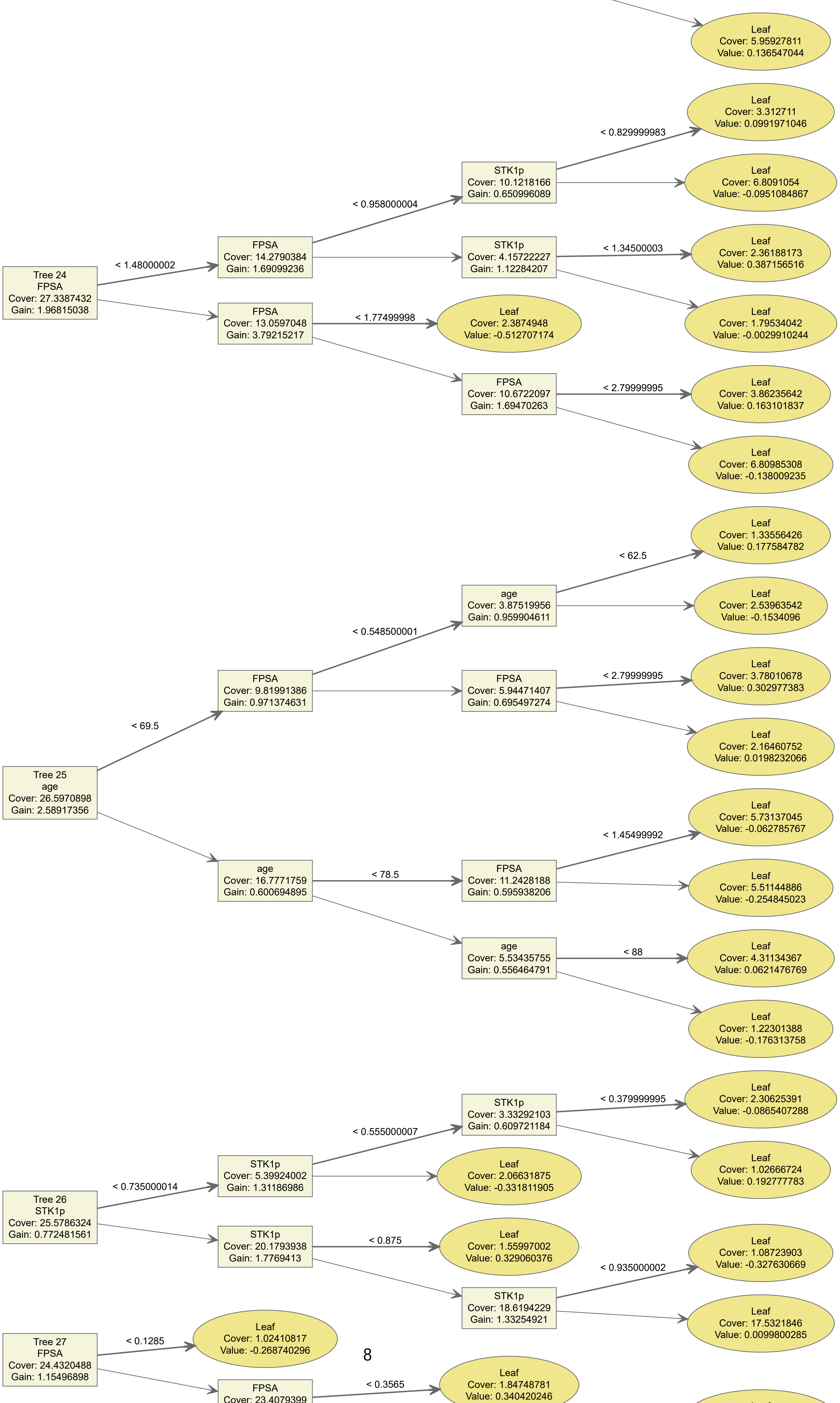
Leaf
Cover: 5.95927811
Value: 0.136547044
Tree 24
FPSA
Cover: 27.3387432
Gain: 1.96815038
< 1.48000002
FPSA
Cover: 14.2790384
Gain: 1.69099236
< 0.958000004
STK1p
Cover: 10.1218166
Gain: 0.650996089
< 0.829999983
Leaf
Cover: 3.312711
Value: 0.0991971046
Leaf
Cover: 6.8091054
Value: -0.0951084867
STK1p
Cover: 4.15722227
Gain: 1.12284207
< 1.34500003
Leaf
Cover: 2.36188173
Value: 0.387156516
Leaf
Cover: 1.79534042
Value: -0.0029910244
FPSA
Cover: 13.0597048
Gain: 3.79215217
< 1.77499998
Leaf
Cover: 2.3874948
Value: -0.512707174
FPSA
Cover: 10.6722097
Gain: 1.69470263
< 2.79999995
Leaf
Cover: 3.86235642
Value: 0.163101837
Leaf
Cover: 6.80985308
Value: -0.138009235
Tree 25
age
Cover: 26.5970898
Gain: 2.58917356
< 69.5
FPSA
Cover: 9.81991386
Gain: 0.971374631
< 0.548500001
age
Cover: 3.87519956
Gain: 0.959904611
< 62.5
Leaf
Cover: 1.33556426
Value: 0.177584782
Leaf
Cover: 2.53963542
Value: -0.1534096
FPSA
Cover: 5.94471407
Gain: 0.695497274
< 2.79999995
Leaf
Cover: 3.78010678
Value: 0.302977383
Leaf
Cover: 2.16460752
Value: 0.0198232066
age
Cover: 16.7771759
Gain: 0.600694895
< 78.5
FPSA
Cover: 11.2428188
Gain: 0.595938206
< 1.45499992
Leaf
Cover: 5.73137045
Value: -0.062785767
Leaf
Cover: 5.51144886
Value: -0.254845023
age
Cover: 5.53435755
Gain: 0.556464791
< 88
Leaf
Cover: 4.31134367
Value: 0.0621476769
Leaf
Cover: 1.22301388
Value: -0.176313758
Tree 26
STK1p
Cover: 25.5786324
Gain: 0.772481561
< 0.735000014
STK1p
Cover: 5.39924002
Gain: 1.31186986
< 0.555000007
STK1p
Cover: 3.33292103
Gain: 0.609721184
< 0.379999995
Leaf
Cover: 2.30625391
Value: -0.0865407288
Leaf
Cover: 1.02666724
Value: 0.192777783
Leaf
Cover: 2.06631875
Value: -0.331811905
STK1p
Cover: 20.1793938
Gain: 1.7769413
< 0.875
Leaf
Cover: 1.55997002
Value: 0.329060376
STK1p
Cover: 18.6194229
Gain: 1.33254921
< 0.935000002
Leaf
Cover: 1.08723903
Value: -0.327630669
Leaf
Cover: 17.5321846
Value: 0.0099800285
Tree 27
FPSA
Cover: 24.4320488
Gain: 1.15496898
< 0.1285
Leaf
Cover: 1.02410817
Value: -0.268740296
FPSA
Cover: 23.4079399
< 0.3565
Leaf
Cover: 1.84748781
Value: 0.340420246

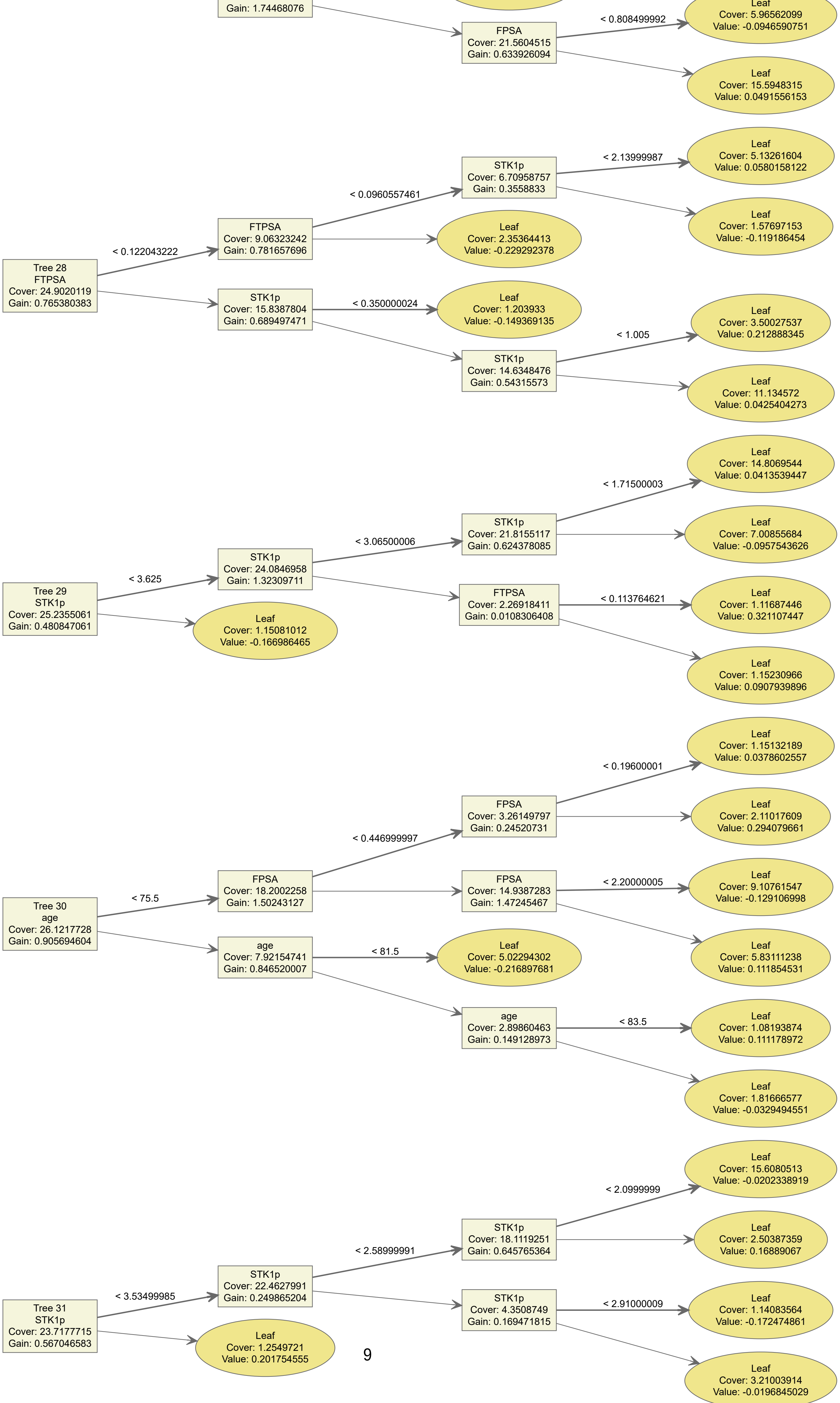
Gain: 1.74468076
FPSA Cover: 21.5604515 Gain: 0.633926094
< 0.808499992
Leaf Cover: 5.96562099 Value: -0.0946590751
Leaf Cover: 15.5948315 Value: 0.0491556153
Tree 28 FTPSA Cover: 24.9020119 Gain: 0.765380383
< 0.122043222
FTPSA Cover: 9.06323242 Gain: 0.781657696
< 0.0960557461
STK1p Cover: 6.70958757 Gain: 0.3558833
< 2.13999987
Leaf Cover: 5.13261604 Value: 0.0580158122
Leaf Cover: 1.57697153 Value: -0.119186454
Leaf Cover: 2.35364413 Value: -0.229292378
STK1p Cover: 15.8387804 Gain: 0.689497471
< 0.350000024
Leaf Cover: 1.203933 Value: -0.149369135
STK1p Cover: 14.6348476 Gain: 0.54315573
< 1.005
Leaf Cover: 3.50027537 Value: 0.212888345
Leaf Cover: 11.134572 Value: 0.0425404273
Tree 29 STK1p Cover: 25.2355061 Gain: 0.480847061
< 3.625
STK1p Cover: 24.0846958 Gain: 1.32309711
< 3.06500006
STK1p Cover: 21.8155117 Gain: 0.624378085
< 1.71500003
Leaf Cover: 14.8069544 Value: 0.0413539447
Leaf Cover: 7.00855684 Value: -0.0957543626
FTPSA Cover: 2.26918411 Gain: 0.0108306408
< 0.113764621
Leaf Cover: 1.11687446 Value: 0.321107447
Leaf Cover: 1.15230966 Value: 0.0907939896
Leaf Cover: 1.15081012 Value: -0.166986465
Tree 30 age Cover: 26.1217728 Gain: 0.905694604
< 75.5
FPSA Cover: 18.2002258 Gain: 1.50243127
< 0.446999997
FPSA Cover: 3.26149797 Gain: 0.24520731
< 0.19600001
Leaf Cover: 1.15132189 Value: 0.0378602557
Leaf Cover: 2.11017609 Value: 0.294079661
FPSA Cover: 14.9387283 Gain: 1.47245467
< 2.20000005
Leaf Cover: 9.10761547 Value: -0.129106998
Leaf Cover: 5.83111238 Value: 0.111854531
age Cover: 7.92154741 Gain: 0.846520007
< 81.5
Leaf Cover: 5.02294302 Value: -0.216897681
age Cover: 2.89860463 Gain: 0.149128973
< 83.5
Leaf Cover: 1.08193874 Value: 0.111178972
Leaf Cover: 1.81666577 Value: -0.0329494551
Tree 31 STK1p Cover: 23.7177715 Gain: 0.567046583
< 3.53499985
STK1p Cover: 22.4627991 Gain: 0.249865204
< 2.58999991
STK1p Cover: 18.1119251 Gain: 0.645765364
< 2.0999999
Leaf Cover: 15.6080513 Value: -0.0202338919
Leaf Cover: 2.50387359 Value: 0.16889067
STK1p Cover: 4.3508749 Gain: 0.169471815
< 2.91000009
Leaf Cover: 1.14083564 Value: -0.172474861
Leaf Cover: 3.21003914 Value: -0.0196845029
Leaf Cover: 1.2549721 Value: 0.201754555

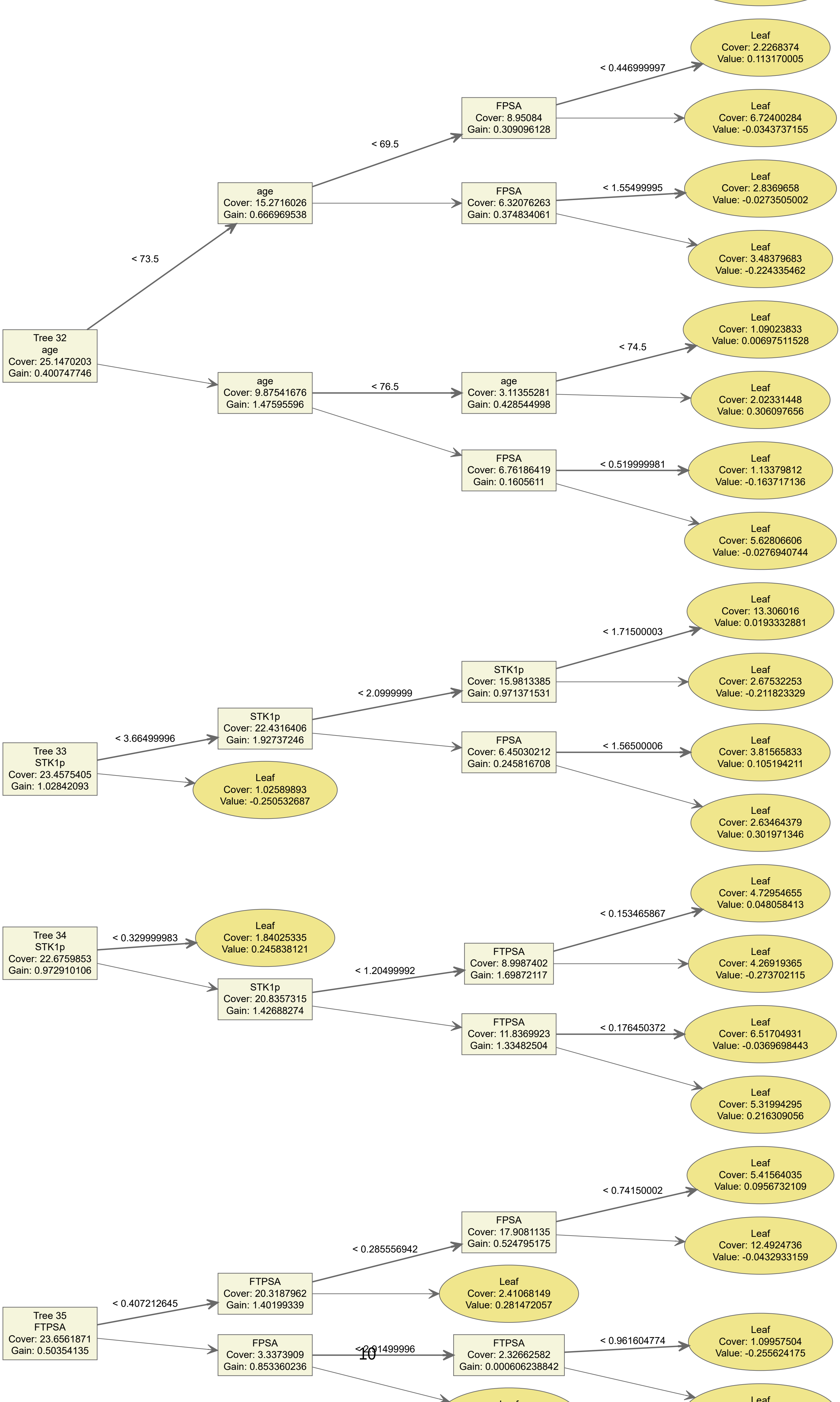

Tree 32
age
Cover: 25.1470203
Gain: 0.400747746
< 73.5
age
Cover: 15.2716026
Gain: 0.666969538
< 69.5
FPSA
Cover: 8.95084
Gain: 0.309096128
< 0.446999997
Leaf
Cover: 2.2268374
Value: 0.113170005
Leaf
Cover: 6.72400284
Value: -0.0343737155
FPSA
Cover: 6.32076263
Gain: 0.374834061
< 1.55499995
Leaf
Cover: 2.8369658
Value: -0.0273505002
Leaf
Cover: 3.48379683
Value: -0.224335462
age
Cover: 9.87541676
Gain: 1.47595596
< 76.5
age
Cover: 3.11355281
Gain: 0.428544998
< 74.5
Leaf
Cover: 1.09023833
Value: 0.00697511528
Leaf
Cover: 2.02331448
Value: 0.306097656
FPSA
Cover: 6.76186419
Gain: 0.1605611
< 0.519999981
Leaf
Cover: 1.13379812
Value: -0.163717136
Leaf
Cover: 5.62806606
Value: -0.0276940744
Tree 33
STK1p
Cover: 23.4575405
Gain: 1.02842093
< 3.66499996
STK1p
Cover: 22.4316406
Gain: 1.92737246
< 2.0999999
STK1p
Cover: 15.9813385
Gain: 0.971371531
< 1.71500003
Leaf
Cover: 13.306016
Value: 0.0193332881
Leaf
Cover: 2.67532253
Value: -0.211823329
FPSA
Cover: 6.45030212
Gain: 0.245816708
< 1.56500006
Leaf
Cover: 3.81565833
Value: 0.105194211
Leaf
Cover: 2.63464379
Value: 0.301971346
Leaf
Cover: 1.02589893
Value: -0.250532687
Tree 34
STK1p
Cover: 22.6759853
Gain: 0.972910106
< 0.329999983
Leaf
Cover: 1.84025335
Value: 0.245838121
STK1p
Cover: 20.8357315
Gain: 1.42688274
< 1.20499992
FTPSA
Cover: 8.9987402
Gain: 1.69872117
< 0.153465867
Leaf
Cover: 4.72954655
Value: 0.048058413
Leaf
Cover: 4.26919365
Value: -0.273702115
FTPSA
Cover: 11.8369923
Gain: 1.33482504
< 0.176450372
Leaf
Cover: 6.51704931
Value: -0.0369698443
Leaf
Cover: 5.31994295
Value: 0.216309056
Tree 35
FTPSA
Cover: 23.6561871
Gain: 0.50354135
< 0.407212645
FTPSA
Cover: 20.3187962
Gain: 1.40199339
< 0.285556942
FPSA
Cover: 17.9081135
Gain: 0.524795175
< 0.74150002
Leaf
Cover: 5.41564035
Value: 0.0956732109
Leaf
Cover: 12.4924736
Value: -0.0432933159
Leaf
Cover: 2.41068149
Value: 0.281472057
FPSA
Cover: 3.3373909
Gain: 0.853360236
< 2.01499996
FTPSA
Cover: 2.32662582
Gain: 0.000606238842
< 0.961604774
Leaf
Cover: 1.09957504
Value: -0.255624175

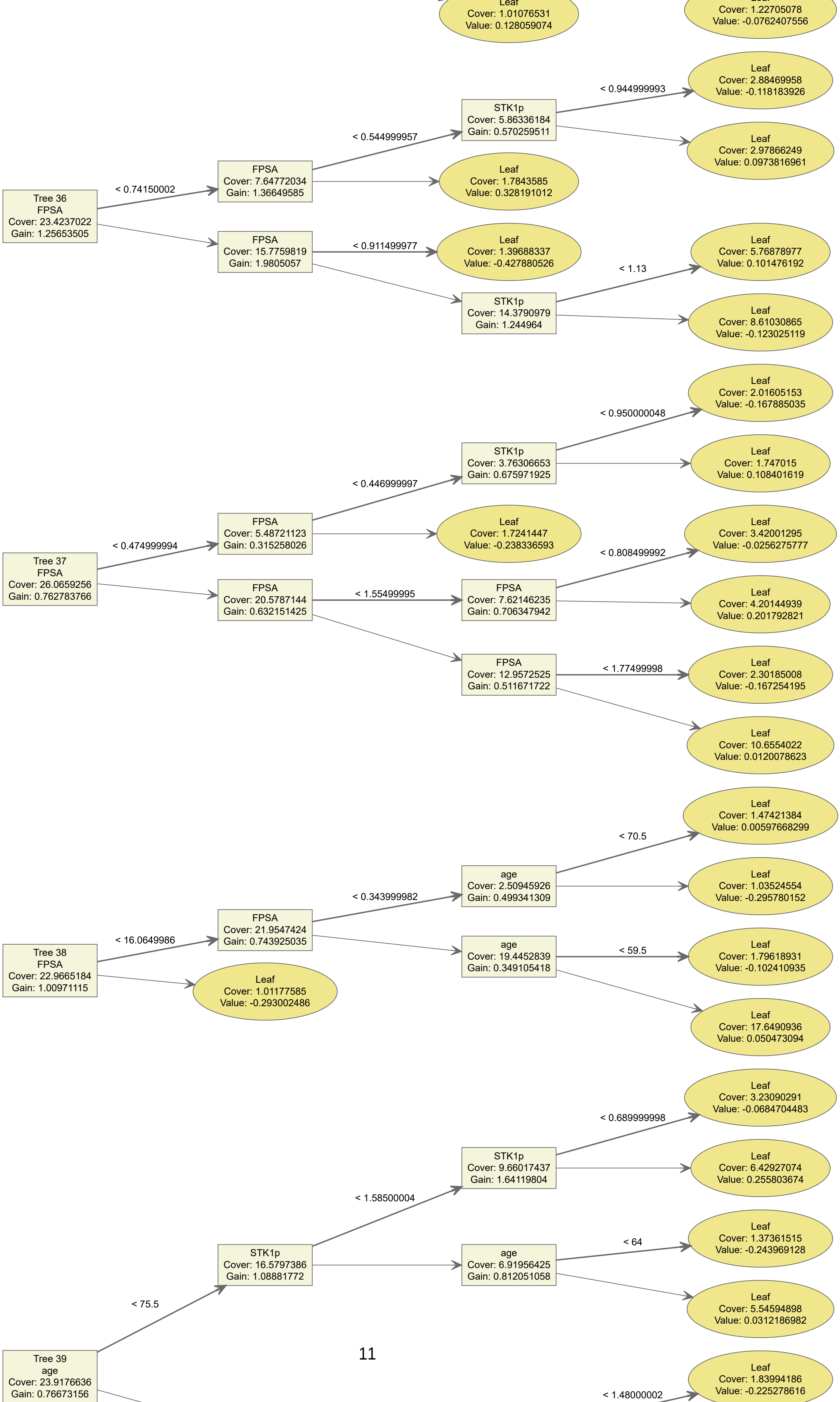
Leaf
Cover: 1.01076531
Value: 0.128059074
Leaf
Cover: 1.22705078
Value: -0.0762407556
Tree 36
FPSA
Cover: 23.4237022
Gain: 1.25653505
< 0.74150002
FPSA
Cover: 7.64772034
Gain: 1.36649585
< 0.544999957
STK1p
Cover: 5.86336184
Gain: 0.570259511
< 0.944999993
Leaf
Cover: 2.88469958
Value: -0.118183926
Leaf
Cover: 2.97866249
Value: 0.0973816961
Leaf
Cover: 1.7843585
Value: 0.328191012
FPSA
Cover: 15.7759819
Gain: 1.9805057
< 0.911499977
Leaf
Cover: 1.39688337
Value: -0.427880526
STK1p
Cover: 14.3790979
Gain: 1.244964
< 1.13
Leaf
Cover: 5.76878977
Value: 0.101476192
Leaf
Cover: 8.61030865
Value: -0.123025119
Tree 37
FPSA
Cover: 26.0659256
Gain: 0.762783766
< 0.474999994
FPSA
Cover: 5.48721123
Gain: 0.315258026
< 0.446999997
STK1p
Cover: 3.76306653
Gain: 0.675971925
< 0.950000048
Leaf
Cover: 2.01605153
Value: -0.167885035
Leaf
Cover: 1.747015
Value: 0.108401619
Leaf
Cover: 1.7241447
Value: -0.238336593
FPSA
Cover: 20.5787144
Gain: 0.632151425
< 1.55499995
FPSA
Cover: 7.62146235
Gain: 0.706347942
< 0.808499992
Leaf
Cover: 3.42001295
Value: -0.0256275777
Leaf
Cover: 4.20144939
Value: 0.201792821
FPSA
Cover: 12.9572525
Gain: 0.511671722
< 1.77499998
Leaf
Cover: 2.30185008
Value: -0.167254195
Leaf
Cover: 10.6554022
Value: 0.0120078623
Tree 38
FPSA
Cover: 22.9665184
Gain: 1.00971115
< 16.0649986
FPSA
Cover: 21.9547424
Gain: 0.743925035
< 0.343999982
age
Cover: 2.50945926
Gain: 0.499341309
< 70.5
Leaf
Cover: 1.47421384
Value: 0.00597668299
Leaf
Cover: 1.03524554
Value: -0.295780152
age
Cover: 19.4452839
Gain: 0.349105418
< 59.5
Leaf
Cover: 1.79618931
Value: -0.102410935
Leaf
Cover: 17.6490936
Value: 0.050473094
Leaf
Cover: 1.01177585
Value: -0.293002486
Tree 39
age
Cover: 23.9176636
Gain: 0.76673156
< 75.5
STK1p
Cover: 16.5797386
Gain: 1.08881772
< 1.58500004
STK1p
Cover: 9.66017437
Gain: 1.64119804
< 0.689999998
Leaf
Cover: 3.23090291
Value: -0.0684704483
Leaf
Cover: 6.42927074
Value: 0.255803674
age
Cover: 6.91956425
Gain: 0.812051058
< 64
Leaf
Cover: 1.37361515
Value: -0.243969128
Leaf
Cover: 5.54594898
Value: 0.0312186982
< 1.48000002
Leaf
Cover: 1.83994186
Value: -0.225278616

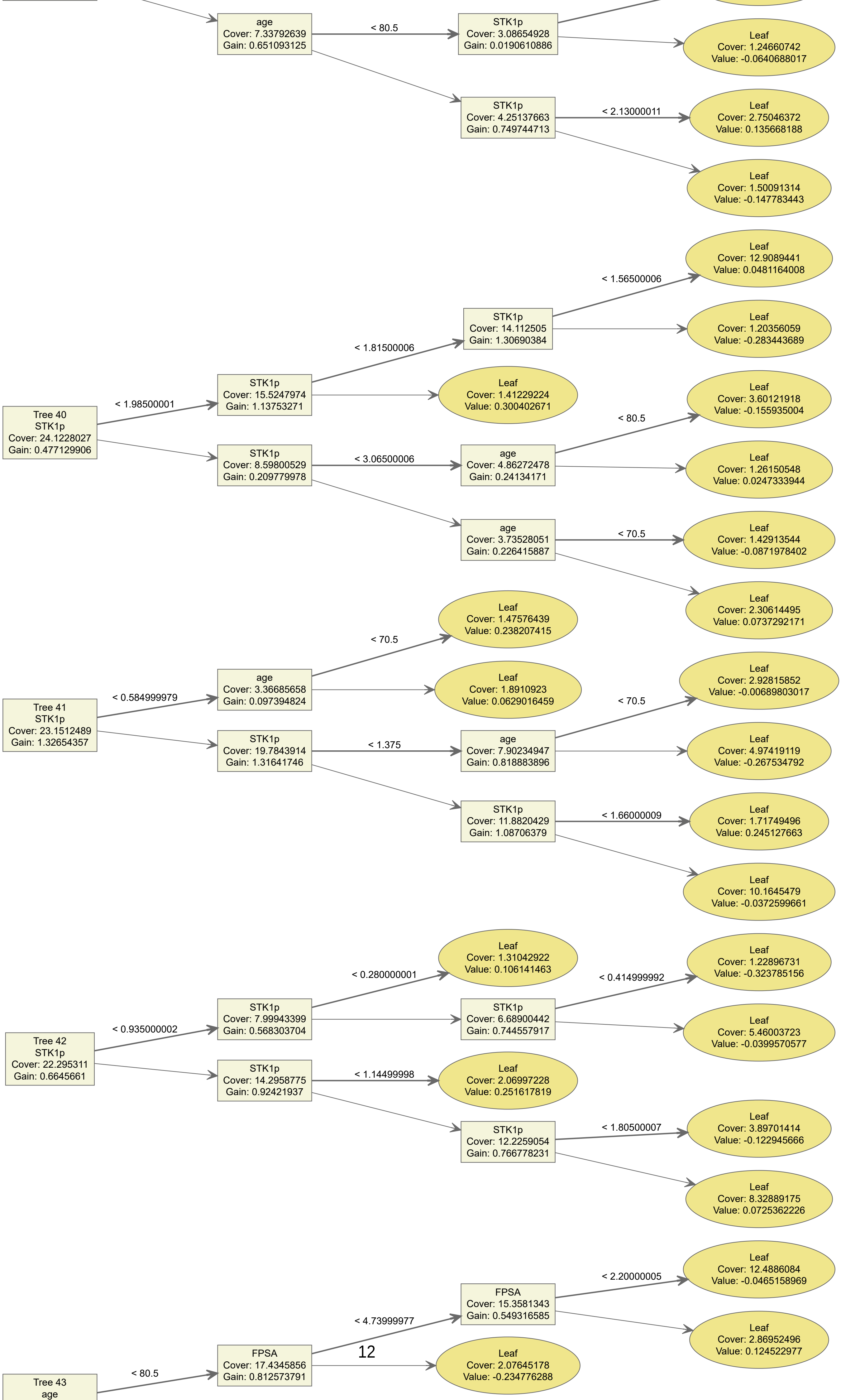
age
Cover: 7.33792639
Gain: 0.651093125
< 80.5
STK1p
Cover: 3.08654928
Gain: 0.0190610886
Leaf
Cover: 1.24660742
Value: -0.0640688017
STK1p
Cover: 4.25137663
Gain: 0.749744713
< 2.13000011
Leaf
Cover: 2.75046372
Value: 0.135668188
Leaf
Cover: 1.50091314
Value: -0.147783443
Tree 40
STK1p
Cover: 24.1228027
Gain: 0.477129906
< 1.98500001
STK1p
Cover: 15.5247974
Gain: 1.13753271
< 1.81500006
STK1p
Cover: 14.112505
Gain: 1.30690384
< 1.56500006
Leaf
Cover: 12.9089441
Value: 0.0481164008
Leaf
Cover: 1.20356059
Value: -0.283443689
Leaf
Cover: 1.41229224
Value: 0.300402671
STK1p
Cover: 8.59800529
Gain: 0.209779978
< 3.06500006
age
Cover: 4.86272478
Gain: 0.24134171
< 80.5
Leaf
Cover: 3.60121918
Value: -0.155935004
Leaf
Cover: 1.26150548
Value: 0.0247333944
age
Cover: 3.73528051
Gain: 0.226415887
< 70.5
Leaf
Cover: 1.42913544
Value: -0.0871978402
Leaf
Cover: 2.30614495
Value: 0.0737292171
Tree 41
STK1p
Cover: 23.1512489
Gain: 1.32654357
< 0.584999979
age
Cover: 3.36685658
Gain: 0.097394824
< 70.5
Leaf
Cover: 1.47576439
Value: 0.238207415
Leaf
Cover: 1.8910923
Value: 0.0629016459
STK1p
Cover: 19.7843914
Gain: 1.31641746
< 1.375
age
Cover: 7.90234947
Gain: 0.818883896
< 70.5
Leaf
Cover: 2.92815852
Value: -0.00689803017
Leaf
Cover: 4.97419119
Value: -0.267534792
STK1p
Cover: 11.8820429
Gain: 1.08706379
< 1.66000009
Leaf
Cover: 1.71749496
Value: 0.245127663
Leaf
Cover: 10.1645479
Value: -0.0372599661
Tree 42
STK1p
Cover: 22.295311
Gain: 0.6645661
< 0.935000002
STK1p
Cover: 7.99943399
Gain: 0.568303704
< 0.280000001
Leaf
Cover: 1.31042922
Value: 0.106141463
STK1p
Cover: 6.68900442
Gain: 0.744557917
< 0.414999992
Leaf
Cover: 1.22896731
Value: -0.323785156
Leaf
Cover: 5.46003723
Value: -0.0399570577
STK1p
Cover: 14.2958775
Gain: 0.92421937
< 1.14499998
Leaf
Cover: 2.06997228
Value: 0.251617819
STK1p
Cover: 12.2259054
Gain: 0.766778231
< 1.80500007
Leaf
Cover: 3.89701414
Value: -0.122945666
Leaf
Cover: 8.32889175
Value: 0.0725362226
Tree 43
age
< 80.5
FPSA
Cover: 17.4345856
Gain: 0.812573791
< 4.73999977
FPSA
Cover: 15.3581343
Gain: 0.549316585
< 2.20000005
Leaf
Cover: 12.4886084
Value: -0.0465158969
Leaf
Cover: 2.86952496
Value: 0.124522977
Leaf
Cover: 2.07645178
Value: -0.234776288

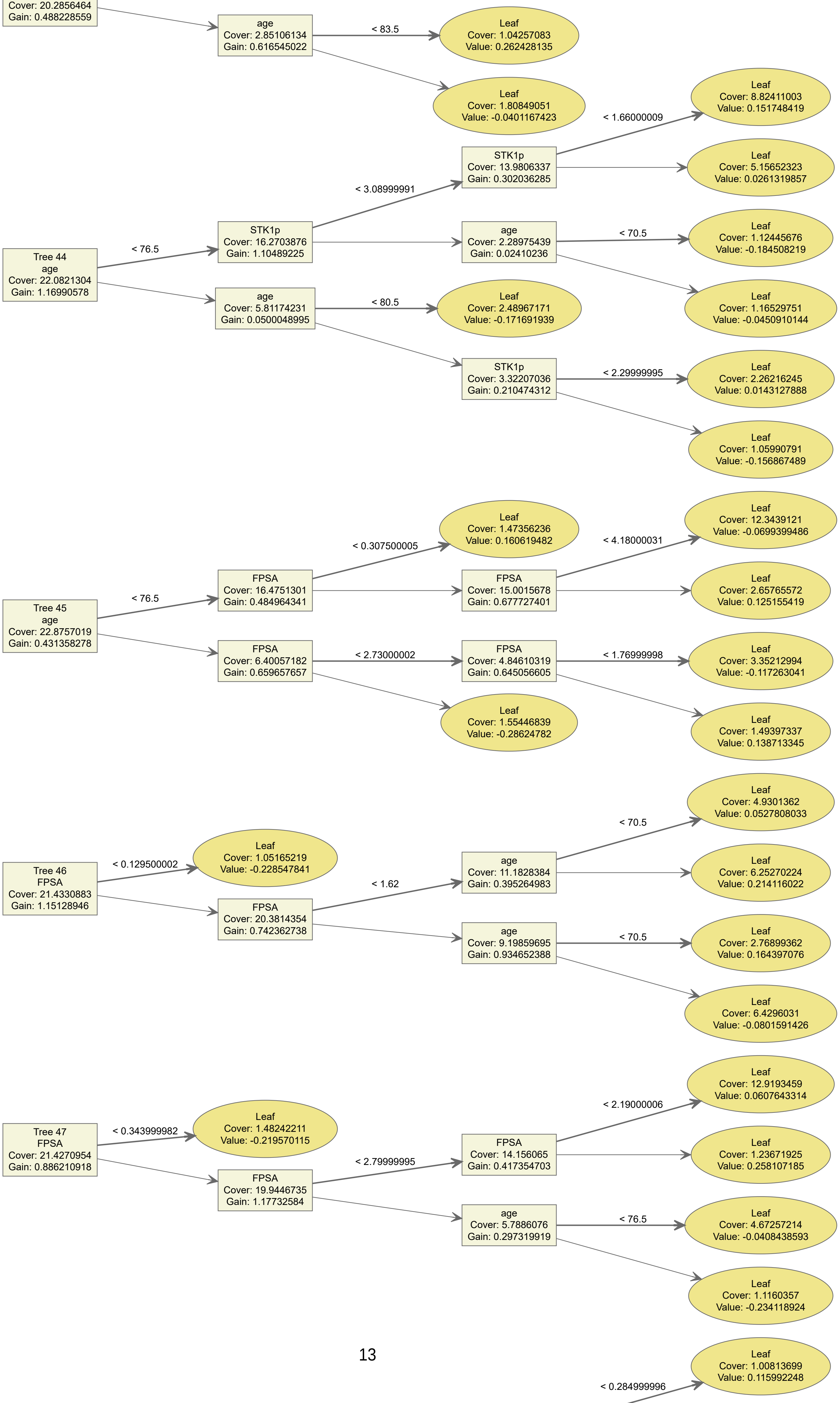
Cover: 20.2856464
Gain: 0.488228559
age
Cover: 2.85106134
Gain: 0.616545022
< 83.5
Leaf
Cover: 1.04257083
Value: 0.262428135
Leaf
Cover: 1.80849051
Value: -0.0401167423
Tree 44
age
Cover: 22.0821304
Gain: 1.16990578
< 76.5
STK1p
Cover: 16.2703876
Gain: 1.10489225
< 3.08999991
STK1p
Cover: 13.9806337
Gain: 0.302036285
< 1.66000009
Leaf
Cover: 8.82411003
Value: 0.151748419
Leaf
Cover: 5.15652323
Value: 0.0261319857
age
Cover: 2.28975439
Gain: 0.02410236
< 70.5
Leaf
Cover: 1.12445676
Value: -0.184508219
Leaf
Cover: 1.16529751
Value: -0.0450910144
age
Cover: 5.81174231
Gain: 0.0500048995
< 80.5
Leaf
Cover: 2.48967171
Value: -0.171691939
STK1p
Cover: 3.32207036
Gain: 0.210474312
< 2.29999995
Leaf
Cover: 2.26216245
Value: 0.0143127888
Leaf
Cover: 1.05990791
Value: -0.156867489
Tree 45
age
Cover: 22.8757019
Gain: 0.431358278
< 76.5
FPSA
Cover: 16.4751301
Gain: 0.484964341
< 0.307500005
Leaf
Cover: 1.47356236
Value: 0.160619482
FPSA
Cover: 15.0015678
Gain: 0.677727401
< 4.18000031
Leaf
Cover: 12.3439121
Value: -0.0699399486
Leaf
Cover: 2.65765572
Value: 0.125155419
FPSA
Cover: 6.40057182
Gain: 0.659657657
< 2.73000002
FPSA
Cover: 4.84610319
Gain: 0.645056605
< 1.76999998
Leaf
Cover: 3.35212994
Value: -0.117263041
Leaf
Cover: 1.49397337
Value: 0.138713345
Leaf
Cover: 1.55446839
Value: -0.28624782
Tree 46
FPSA
Cover: 21.4330883
Gain: 1.15128946
< 0.129500002
Leaf
Cover: 1.05165219
Value: -0.228547841
FPSA
Cover: 20.3814354
Gain: 0.742362738
< 1.62
age
Cover: 11.1828384
Gain: 0.395264983
< 70.5
Leaf
Cover: 4.9301362
Value: 0.0527808033
Leaf
Cover: 6.25270224
Value: 0.214116022
age
Cover: 9.19859695
Gain: 0.934652388
< 70.5
Leaf
Cover: 2.76899362
Value: 0.164397076
Leaf
Cover: 6.4296031
Value: -0.0801591426
Tree 47
FPSA
Cover: 21.4270954
Gain: 0.886210918
< 0.343999982
Leaf
Cover: 1.48242211
Value: -0.219570115
FPSA
Cover: 19.9446735
Gain: 1.17732584
< 2.79999995
FPSA
Cover: 14.156065
Gain: 0.417354703
< 2.19000006
Leaf
Cover: 12.9193459
Value: 0.0607643314
Leaf
Cover: 1.23671925
Value: 0.258107185
age
Cover: 5.7886076
Gain: 0.297319919
< 76.5
Leaf
Cover: 4.67257214
Value: -0.0408438593
Leaf
Cover: 1.1160357
Value: -0.234118924
< 0.284999996
Leaf
Cover: 1.00813699
Value: 0.115992248

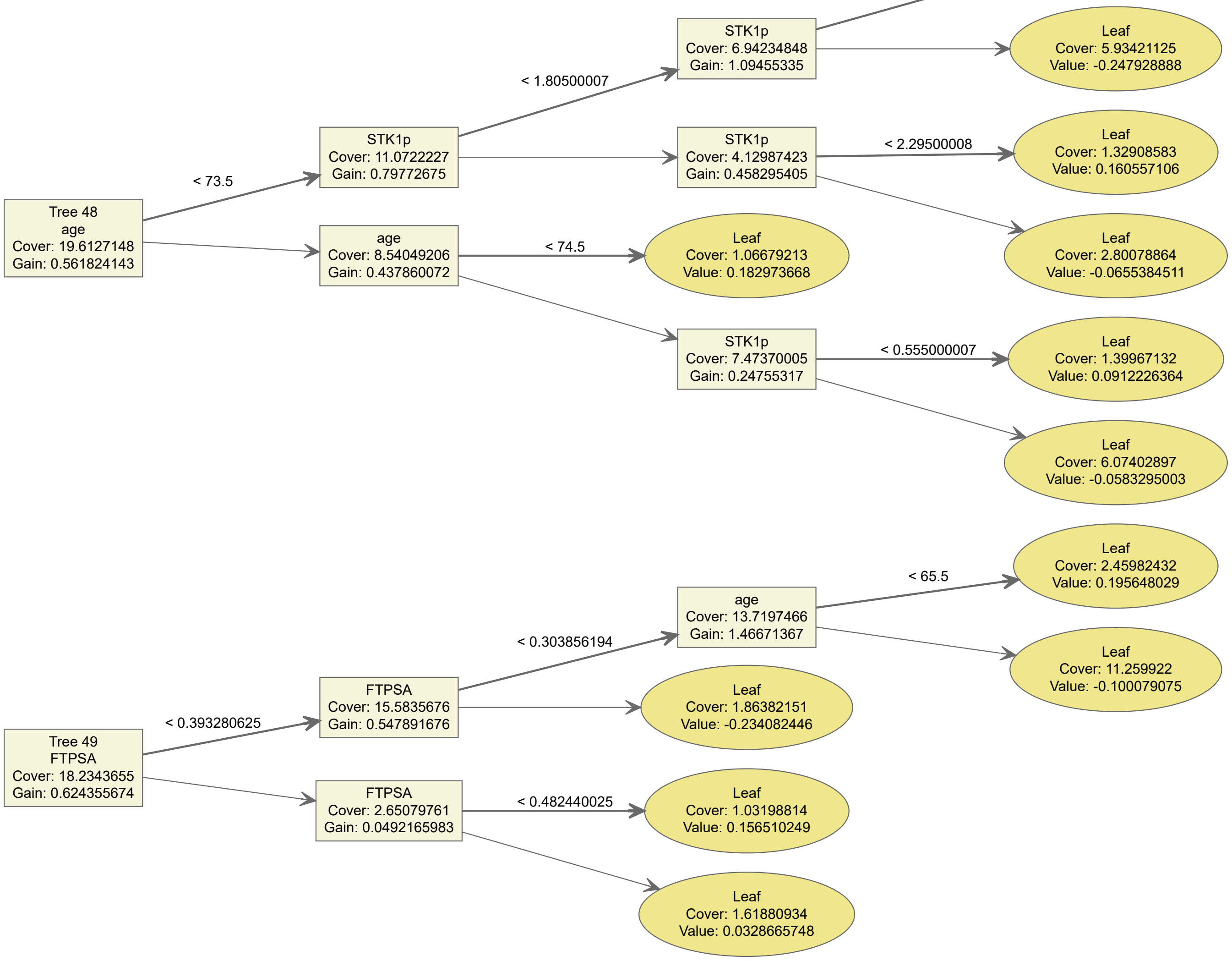

Supplementary Fig 1. All decision trees of the XGBOOST model.
